# Parkinson’s disease-associated LRRK2-G2019S mutant acts through regulation of SERCA activity to control ER stress in astrocytes

**DOI:** 10.1186/s40478-019-0716-4

**Published:** 2019-05-02

**Authors:** Jee Hoon Lee, Ji-hye Han, Hyunmi Kim, Sang Myun Park, Eun-hye Joe, Ilo Jou

**Affiliations:** Department of Pharmacology, and Chronic Inflammatory Disease Research Center, Ajou University School of Medicine, Suwon, 16499 Korea

**Keywords:** Parkinson’s disease, LRRK2-G2019S, ER stress, SERCA, Mitochondria, Astrocytes

## Abstract

Accumulating evidence indicates that endoplasmic reticulum (ER) stress is a common feature of Parkinson’s disease (PD) and further suggests that several PD-related genes are responsible for ER dysfunction. However, the underlying mechanisms are largely unknown. Here, we defined the mechanism by which LRRK2-G2019S (LRRK2-GS), a pathogenic mutation in the PD-associated gene LRRK2, accelerates ER stress and cell death. Treatment of cells with α-synuclein increased the expression of ER stress proteins and subsequent cell death in LRRK2-GS astrocytes. Intriguingly, we found that LRRK2-GS localizes to the ER membrane, where it interacts with sarco/endoplasmic reticulum Ca^2+^-ATPase (SERCA) and suppress its activity by preventing displacement of phospholamban (PLN). LRRK2-GS–mediated SERCA malfunction leads to ER Ca^2+^ depletion, which induces the formation of mitochondria-ER contacts and subsequent Ca^2+^ overload in mitochondria, ultimately resulting in mitochondrial dysfunction. Collectively, our data suggest that, in astrocytes, LRRK2-GS impairs ER Ca^2+^ homeostasis, which determines cell survival, and as a result, could contribute to the development of PD.

**Electronic supplementary material:**

The online version of this article (10.1186/s40478-019-0716-4) contains supplementary material, which is available to authorized users.

## Introduction

Parkinson’s disease (PD), the second-most common neurodegenerative disease, is characterized by selective loss of dopaminergic (DA) neurons of the substantia nigra pars compacta (SNpc), accumulation of intracellular inclusions containing α-synuclein, and subsequent progressive impairment of dopaminergic neurons—the clinical feature of PD [6, 58]. It is mostly sporadic; less than 10% of PD cases are inherited [28]. Despite intensive investigation, current treatments, medications, and even surgery do not cure or stop the progression of PD, highlighting the critical importance of understanding the mechanisms involved in the loss of dopaminergic neurons in PD. Recent studies suggested that non-neuronal cells, such as astrocytes, accumulate α-synuclein during PD, and can contribute to neurodegeneration through various pathways [7, 56], suggesting astrocytes play critical roles in neuronal dysfunction and interplays between astrocytes and neurons may provide insights into neuronal dysfunction and death in PD. However, relationship between astrocytic α-synucleintoxicity and neuronal dysfunction remains unclear.

Accumulating evidence from studies of the human PD brain suggest that the progressive deterioration of vulnerable SNpc DA neurons arises from cellular disturbances produced by misfolding and aggregation of the synaptic protein α-synuclein, disruption of the autophagy-lysosome system, mitochondrial dysfunction, and/or endoplasmic reticulum (ER) stress [31]. The ER is the subcellular site of protein folding and maturation, and the main intracellular Ca^2+^ store of the cell. Since ER resident chaperones are involved in protein folding require high Ca^2+^ concentrations for their activity, altered ER Ca^2+^ homeostasis can result in an imbalance between the capacity of the protein processing machinery and the amount of unfolded proteins requiring processing, leading to an accumulation of unfolded proteins and ER stress [24, 30].

The increasing number of unfolded proteins inside the ER lumen provokes the dissociation of grp78/BiP (78 kDa glucose-regulated protein) from three ER transmembrane receptors—PKR-like endoplasmic reticulum kinase (PERK), activating transcription factor 6 (ATF6) and inositol-requiring enzyme 1 (IRE1)—thereby initiating the unfolded protein response (UPR). This ER-specific stress response serves to maintain cell survival through different molecular pathways, including reduced translation-initiation rate, enhanced protein folding, and/or elimination of misfolded proteins [53]. Under conditions of prolonged or severe ER stress, however, the UPR switches from homeostatic feedback regulation towards proapoptotic signaling [52]. Growing evidence from recent studies indicates that the accumulation of misfolded proteins in the brain is a salient feature of most neurodegenerative diseases, including Alzheimer’s disease, amyotrophic lateral sclerosis, Huntington’s disease, and PD [29]. These diseases are now classified as protein misfolding disorders (PMDs) [13, 57]. The mechanisms leading to ER stress in PMDs and the actual impact of the UPR on the degeneration cascade in this disease are just starting to be uncovered.

A major genetic form of PD is caused by mutations in leucine-rich repeat kinase 2 protein (LRRK2) [37, 66]. The G2019S (GS) mutation within the kinase domain encoded by exon 41 is the most common mutation of LRRK2, which alters LRRK2 GTPase and kinase activities and accounts for ~ 1% of sporadic PD and up to 25% of familial PD in certain populations [28]. The LRRK2 G2019S mutation causes a gain of function effect that could involve alterations in autophagy-lysosomal [61] and microRNA [10] pathways, and dysregulation of protein quality control [18, 32], oxidative stress [32, 49], and protein synthesis [16] mechanisms. Although the function of LRRK2 in ER stress remains a matter of debate, studies in *Caenorhabditis elegans* have demonstrated that expression of wild-type LRKK2 protects dopaminergic neurons against neurotoxicity induced by human α-synuclein through upregulation of grp78/BiP [65]. Another study using a *C. elegans* model lacking the LRRK2 homolog suggested that LRRK2 is critical for preventing ER stress and spontaneous neurodegeneration [50]. It has also been suggested that LRRK2 regulates anterograde ER-Golgi transport by anchoring Sec16A at ER exit sites, leading to a reduction in ER stress [3]. Despite these interesting findings, the possible contribution of ER stress to the pathogenic manifestations of mutant LRKK2 in mammalian cells has not yet been addressed.

In this study, we showed that the LRRK2 G2019S mutant is responsible for ER stress in α-synuclein–treated brain astrocytes. Immunostaining and subcellular fractionation revealed that LRRK2-G2019S dissociates from 14 to 3-3 s and then localizes to the ER membrane. Using mass spectrometry (MS) proteomic screening of LRRK2-associated proteins, we identified sarco/endoplasmic reticulum Ca^2+^-ATPase (SERCA) as a protein that strongly interacts with the LRRK2 G2019S mutant in the ER. Binding of LRRK2 G2019S to SERCA inactivated SERCA by maintaining the interaction of SERCA with phospholamban (PLN), which negatively regulates SERCA activity through direct association. The inactivation of SERCA by LRRK2 G2019S led to ER Ca^2+^ depletion, followed by chronic ER stress, mitochondria dysfunction, and cell death. Collectively, these findings indicate that LRRK2-G2019S accelerates ER stress, suggesting a molecular basis for the pathogenesis of PD in patients harboring this mutant.

## Materials and methods

### Animals

G2019S-*LRRK2*-Tg FVB mice were purchased from Jackson Laboratory (stock #009609, Bar Harbor, ME, USA). Non-Tg and G2019S-*LRRK2*-Tg heterozygous mice were prepared by crossing G2019S-*LRRK2* heterozygous mice with wild-type mice. Genotyping was carried according to the vendor’s instructions. All animal procedures were approved by the Ajou University Institutional Animal Experimentation Committee (AMC-119).

### Cell culture

Primary astrocytes were cultured from the cerebral cortices of 1-d-old non-Tg and G2019S-*LRRK2*-Tg heterozygous mice. Briefly, cortices were triturated into single cells in Dulbecco’s modified Eagle’s medium (DMEM; Sigma-Aldrich, St. Louis, MO, USA) containing 10% (*v*/v) fetal bovine serum (FBS; Hyclone, South Logan, UT, USA), plated into 75 cm^2^ T-flasks, and incubated for 2 wk. Following removal of microglia, primary astrocytes were enzymatically dissociated with trypsin (Sigma) for 5 min at 37 °C in a humidified 5% CO_2_, 95% air chamber. Trypsinization was quenched by adding astrocyte culture medium and centrifuged (~ 200 g) for 5 min. Microglia and meningeal cells were depleted by incubating astrocytes with serum-free DMEM for 2 d before use. The cell populations obtained consisted of more than 95% authentic astrocytes, as determined using the astrocyte marker GFAP (glial fibrillary acidic protein) immunofluorescence.

Primary neurons were cultured from embryonic mouse cortices (E17). Briefly, cortices were dissected in Hank’s Buffered Salt Solution (HBSS; Gibco, Carlsbad, CA, USA) supplemented with HEPES (10 mM, pH 7.4). Tissues were incubated in HBSS containing trypsin (Gibco) and DNase I (100 μg/ml) for 15 min at 37 °C, then dissociated by gentle pipetting. Dissociated cells were plated in poly-D-lysine (1 mg/ml)-coated 6-well plate (3 × 10^5^ cells/well) or 12-mm cover glasses (1 × 10^4^ cells) in Neurobasal medium containing B27 (2%), sodium pyruvate (1%), penicillin/streptomycin (1%), and GlutaMax (1%) (all supplements were from Gibco). Cells were incubated for 10 d and then challenged with α-synuclein for 24–48 h. For co-culture with astrocytes, cortical neurons were seeded on non-Tg or LRRK2-GS astrocyte monolayers for 10 d, then treated with α-synuclein for 24–48 h and assayed by immunocytochemistry.

C2 myoblasts were cultured as previously described [22]. Briefly, cells were cultured in DMEM supplemented with 10% (*v*/v) FBS. Myogenic differentiation was induced by incubating in low-serum medium (DMEM plus 1% FBS) for 3 d. To induce ER stress, the astrocytes, neuron, and C2 myoblasts were incubated with vehicle or tunicamycin (0.1 μg/mL) (Sigma) for 3 ~ 24 h for each separate experiment.

### Organotypic brain slice culture

Organotypic brain slice cultures were prepared from postnatal day 7–8 non-Tg and LRRK2-GS mice. Mice were anesthetized, and cortices were dissected and coronally sectioned (400 μm) using a McIlwain tissue chopper (Mickle Laboratory Engineering, UK). Slices were mounted on Millicell cell culture inserts (0.4 μm pore size, 30 mm diameter; Millipore, Burlington, MA, USA). Culture medium (50% minimal essential medium [MEM] containing 25% HBSS, 25% heat-inactivated horse serum, 0.5% glucose, 1 mM l-glutamine) was changed every 2–3 d. Slices were treated with α-synuclein or tunicamycin after 7 d in culture.

### α-Synuclein

The human recombinant α-synuclein was purchased from rPeptide (Watkinsville, GA, USA) or was a kind gift from Professor Sang Myun Park (Ajou University, Korea). Purified recombinant α-synuclein protein was stored at − 80 °C until use as monomeric α-synuclein. The α-synuclein oligomer was prepared as previously described [4] with minor modifications. Briefly, 1 mg/ml of monomeric α-synuclein was dissolved in PBS (0.01 M sodium phosphate (pH 7.4), 150 mM NaCl), and incubated at 37 °C with continuous agitation at 250 rpm for 2 d, then stored at − 80 °C until use as oligomeric α-synuclein. Monomeric or oligomeric α-synuclein was mixed with 20 μM thioflavin T in 5x assay buffer (250 mM glycine, pH 8.5) in a final volume of 200 μl, and fluorescence was measured at 482 nm with excitation at 446 nm using a PerkinElmer Vitor3 multiplate reader (PerkinElmer, Waltham, MA, USA). Also, a 20 μl aliquot of α-synuclein was adsorbed onto carbon-coated copper grid and air-dried for 10 min. After negative staining with 2% uranyl acetate for another 10 min, α-synuclein was observed with an electron microscope (SIGMA500, Zeiss, Germany).

### Plasmid constructs

Plasmid DNA for C-terminal 3xMyc-tagged wild-type LRRK2, LRRK2-G2019S and LRRK2-D1994A, and FLAG-tagged LRRK2 fragments (L1–L4) were kind gifts from Professor Eun-hye Joe (Ajou University, Korea). Myc-tagged mouse SERCA was purchased from Origene (Rockville, MD, USA). pCMV-G-CEPIA1*er* was a gift from Masamitsu Iino (Addgene plasmid #58215); CMV-R-GECO1*mito* was a gift from Robert Campbell (Addgene plasmid #46021); pEF-myc-ER-E2-Crimson was a gift from Benjamin Glick (Addgene plasmid #38770); and mito-PAGFP was a gift from Richard Youle (Addgene plasmid #23348).

### Subcellular fractionation

HEK293T cells were co-transfected with siRNA targeting the 3′-UTR region of LRRK2 and 3xMyc-tagged wild-type LRRK2 or G2019S-mutated LRRK2. After 24 h, cells were treated with α-synuclein for 24 h. ER, mitochondria, and MAM were isolated from HEK293T cells following the previously described protocols [64] with minor modifications. Briefly, HEK293T cells at ~ 90–100% confluence were harvested from 25 dishes (10 cm) and homogenized in isolation buffer (225 mM mannitol, 75 mM sucrose, 0.1 mM EGTA, 30 mM Tris-HCl pH 7.4) using a Dounce tissue grinder (Wheaton, Millville, NJ, USA). Nuclei and debris were removed by centrifuging the homogenate twice at 600×g for 10 min, after which the collected supernatant was centrifuged for 15 min at 8000×g. The resulting pellet was collected as the crude mitochondrial fraction. The supernatant was centrifuged at 20,000 g for 1 h, then again at 100,000 g for 1 h, after which the resulting pellet was resuspended as the ER fraction. The supernatant was kept as the cytosolic fraction. For pure mitochondria and MAM fractions, the crude mitochondrial pellet was resuspended in 2 ml mitochondrial resuspension buffer (MRB; 250 mM mannitol, 5 mM HEPES pH 7.4, 0.5 mM EGTA), layered over 30% Percoll medium in a centrifuge tube, and centrifuged at 95,000×g for 30 min. The lower layer (pure mitochondria) and intermediate layer (MAM) between the light membrane and pure mitochondria fractions were then collected. The pure mitochondria fraction was diluted in MRB buffer and further centrifuged at 10,000 g, after which the pellet was resuspended in 300 μl of MRB buffer. The MAM fraction was diluted 10x in MRB buffer and centrifuged at 100,000×g for 1 h, after which the pellet was resuspended in a small volume of MRB buffer.

### Time-lapse Ca^2+^ imaging

Astrocytes isolated from non-Tg or LRRK2-GS mice were plated on glass-bottom dishes (Nest Scientific, China) and then treated with α-synuclein or tunicamycin (0.1 μg/ml) for 24 h. ER Ca^2+^ release into the cytosol was measured in cells loaded with 5 μM Fluo4-AM (Invitrogen, Carlsbad, CA, USA) in HBSS containing 0.05% Pluronic F-127 (Thermo Fisher Scientific, Waltham, MA, USA) at 37 °C. After 20 min, excess Fluo4-AM was removed by washing cells twice, and then the wash buffer was replaced with HBSS without Ca^2+^ and Mg^2+^. Cells were stimulated with 500 μM histamine (Sigma) and imaged under a confocal microscope (TCS, DMi8; Leica, Germany) equipped with a 40x objective (NA 1.10) in a chamber with temperature and CO_2_ control and a heated stage (LCI, Korea). Images were captured at a rate of one frame per 1 or 2 s. For evaluate Ca^2+^ dynamics in ER and mitochondria, transfection was performed by incubating astrocytes with a mixture of 1 μg of G-CEPIA1*er* and R-GECO1*mito,* Lipofectamine 2000 (Life Technologies) for 5 h. After incubation, the medium was changed and the cells were incubated at 37 °C in 5% CO_2_. After 24 h, the medium was replaced with HBSS without Ca^2+^ and Mg^2+^, and then cells were stimulated with histamine and imaged with a confocal microscope equipped with a 40x objective (NA 1.10). Images were captured at a rate of one frame per 1 or 2 s with the following excitation/emission spectra: G-CEPIA1*er*, 488 nm/500–550 nm; R-GECO1*mito*, 552 nm/560–800 nm. After all Ca^2+^ intensities were corrected for background subtraction, *Δ*F values were calculated from (F–F_0_). F_0_ values, used for normalization, were defined by averaging 10 frames before stimulation.

### Immunoprecipitation and nanoLC-MS/MS

HEK293T cells were co-transfected with siRNA targeting the 3′-UTR region of LRRK2 and 3xMyc-tagged wild-type LRRK2 or G2019S-mutated LRRK2. After 24 h, cells were treated with α-synuclein for 24 h and then lysates were prepared using a modified RIPA buffer. Lysates (1 mg) were immunoprecipitated with anti-Myc antibody-conjugated magnetic beads (Invitrogen). Immunoprecipitated proteins were digested with trypsin and quantified on a nanoLC-MS/MS platform. The raw MS files were analyzed and searched against a human protein database based on the species of the sample using Proteome Discoverer 2.0 (Thermo Fisher Scientific). The parameters were set as follows: protein modifications, carbamidomethylation (C) (fixed) and oxidation (M) (variable); enzyme specificity, trypsin; maximum missed cleavages, 2; precursor ion mass tolerance, 10 ppm; and MS/MS tolerance, 0.6 Da. To ensure high confidence identifications, peptide-to-spectrum match (PSM), peptides, and proteins were filtered at a less than 1% false discovery rate (FDR). Three independent replicates for each experimental condition were carried out to control for intrasample variation. In total, 2831 proteins were identified for this project, and were divided into two categories of relative quantitation. Those with a quantitative ratio greater than 1.5 were considered up-regulated, whereas those with a quantitative ratio less than 0.67 were considered down-regulated (Creative Proteomics, Shirley, NY, USA). Protein interactions identified in this study have been submitted to the IMEx (http://www.imexconsortium.org) consortium through IntAct [34] and assigned the identifier IM-26684.

### Cytoscape analysis of LRRK2-interacting proteins

LRRK2-interacting proteins identified by nanoLC-MS/MS were analyzed using the ClueGo Cytoscape plugin with Cellular Component and Biological Process function Gene Ontology annotations. The groups consist of nodes (cellular component) connected to reflect functional relationships between nodes. Each functional group is annotated in colored type.

### Analysis of mitochondrial morphology

For visualization of mitochondria, cells were stained with 250 nM MitoTracker Red CMXRos (Invitrogen) for 20 min at 37 °C. Mitochondrial morphology subtypes were quantified using an automated classification system, according to Peng et al. [39]. After semi-automated segmentation of cell micrographs, mitochondria were classified into six distinct subtypes—small globe, swollen globe, straight tubule, twisting tubule, branch tubule and loop—using automated classification software. The proportion of tubular mitochondria was calculated by totaling straight tubule, twisting tubule, and branch tubule mitochondrial populations. The proportion of fragmented tubular mitochondria was calculated based on small globe mitochondrial populations. High-powered (400 x) fields of micrographs from three independent areas per group were analyzed, and about 200–300 mitochondria from 5 cells were used in calculations.

### Tetramethylrhodamine methyl ester imaging

For tetramethylrhodamine methyl ester (TMRM) imaging, astrocytes isolated from non-Tg or LRRK2-GS mice were plated on glass-bottom dishes (Nest Scientific), and then treated with α-synuclein. After 24 h, the medium was replaced with HBSS supplemented with 10 nM TMRM (Invitrogen), and cells were incubated for 30 min at 37 °C. Cells were imaged with a confocal microscope (TCS, DMi8, Leica) equipped with a 40x objective (1.10 NA) in a chamber with temperature and CO_2_ control and a heated stage. Mitochondrial membrane potential was dissipated by treating cells with 10 μM carbonyl cyanide m-chlorophenyl hydrazone (CCCP; Sigma).

### Flow cytometry

Astrocytes isolated from non-Tg and LRRK2-GS mice were treated with α-synuclein for 24–48 h and then stained with the superoxide indicator, MitoSOX Red (5 μM; Invitrogen), for 30 min at 37 °C. Cells were treated with trypsin and analyzed by flow cytometry using a FACS Canto II flow cytometer (BD Biosciences, San Jose, CA, USA).

### Immunofluorescence

Astrocytes isolated from non-Tg and LRRK2-GS mice were treated with α-synuclein for 24 h and then fixed with 4% paraformaldehyde and then permeabilized with 0.25% triton for 10 min. Cells were washed three times in PBS and blocked with 1% BSA for 1 h. Cells were incubated with primary antibodies overnight at 4 °C. Washed cells were incubated with fluorescent secondary antibody (Invitrogen) for 1 h at room temperature. Cover slips were mounted with Vectashield containing DAPI (Vector Laboratories) and viewed on confocal microscope confocal microscope (TCS, DMi8; Leica).

### TUNEL assay

TUNEL assays were carried out using a commercial kit according to the manufacturer’s instructions (Invitrogen). Briefly, cells and proteinase K-treated organotypic slices were rinsed with phosphate-buffered saline (PBS), and then incubated in 1x equilibration buffer for 10 min. Thereafter, samples were incubated with terminal deoxynucleotidyl transferase (TdT) for 1 h at 37 °C, blocked with stop/wash buffer, and incubated with peroxidase antibody for 30 min at room temperature. The images were taken from layer 1 of the cortex in brain slices (*n* = 5 slices from 3 mouse) and the percentage of TUNEL-positive cells was determined in at least 10 optical fields.

### Proximity ligation assay (PLA)

Fixed cells were stained with the indicated rabbit and mouse antibodies. Duolink-PLA (Olink Bioscience, Sweden) procedures were performed according to the manufacturer’s instructions. Each discrete red spot represents a protein-protein complex (radius < 40 nm).

### Proteinase protection assay

ER-enriched fractions from non-Tg and LRRK2-GS astrocytes were prepared using the method described above (refer to Subcellular fractionation). For proteinase protection assays, ER-enriched fractions were diluted in homogenization buffer at a protein concentration of 0.5 μg/μl and incubated in the absence or presence of 150 μg/ml Proteinase K (Invitrogen) for 30 min. The reaction was terminated by adding phenylmethylsulfonyl fluoride (PMSF) to a final concentration of 5 mM and incubating on ice for 5 min. Subsequently, an equal volume of 2x SDS sample buffer was added and protein in the samples was denatured by incubation at 100 °C for 5 min. For Western blot analysis, samples were resolved by sodium dodecyl sulfate-polyacrylamide gel electrophoresis (SDS/PAGE). Digestion was monitored using endogenous calnexin and BiP as controls.

### Synthesis and transfection of siRNA

siRNA duplex oligonucleotides were chemically synthesized by Bioneer (Daejeon, Korea) and Santa Cruz (Santa Cruz, CA). Details of the siRNA sequences are described in Additional file 2: Table S2. Confluent astrocytes were transfected with siRNA oligonucleotides (50 μM) using Lipofectamine RNAiMax reagent (Invitrogen) according to the manufacturer’s instructions. All assays were performed at least 48 h after RNAi transfection.

### Western blotting

Cells were lysed with RIPA buffer (50 mM Tris-HCl pH 7.5, 150 mM NaCl, 1% Triton X-100, 1% sodium deoxycholate, 0.1% SDS, 2 mM EDTA pH 8.0) supplemented with a protease inhibitor cocktail (GenDEPOT, Barker, TX, USA) at 4 °C for 30 min. Samples were separated by SDS-PAGE and transferred to nitrocellulose membranes. Membranes were incubated with primary antibodies (Additional file 2: Table S4) and horseradish peroxidase (HRP)-conjugated secondary antibodies, and immunoreactive proteins were visualized using an enhanced chemiluminescence system (Ab Frontier, Seoul, Korea).

### Real time quantitative reverse transcription-polymerase chain reaction (RT-qPCR) analysis

Total RNA was isolated using RNAiso Plus (TaKaRa, Japan), and cDNA was synthesized using avian myeloblastosis virus reverse transcriptase (New England Biolabs, Ipswich, MA, USA) and oligo (dT) primers (Promega, Madison, WI, USA), according to the manufacturers’ instructions. For qPCR, amplification reactions were performed using a Thermal Cycler Dice Real-Time System (TaKaRa) with SYBR Premix Ex Taq master mix (TaKaRa) according to the manufacturer’s instructions. The primers used for qPCR (Bioneer) are described in Additional file 2: Table S3.

### Statistical analysis

The significance of differences between groups was determined using Student’s *t*-test. A *p-*value of 0.05 was considered significant. Values are presented as means ± standard deviation (SD).

## Results

### α-Synuclein induces ER stress and cell death in LRRK2-G2019S astrocytes

**Fig. 1:**
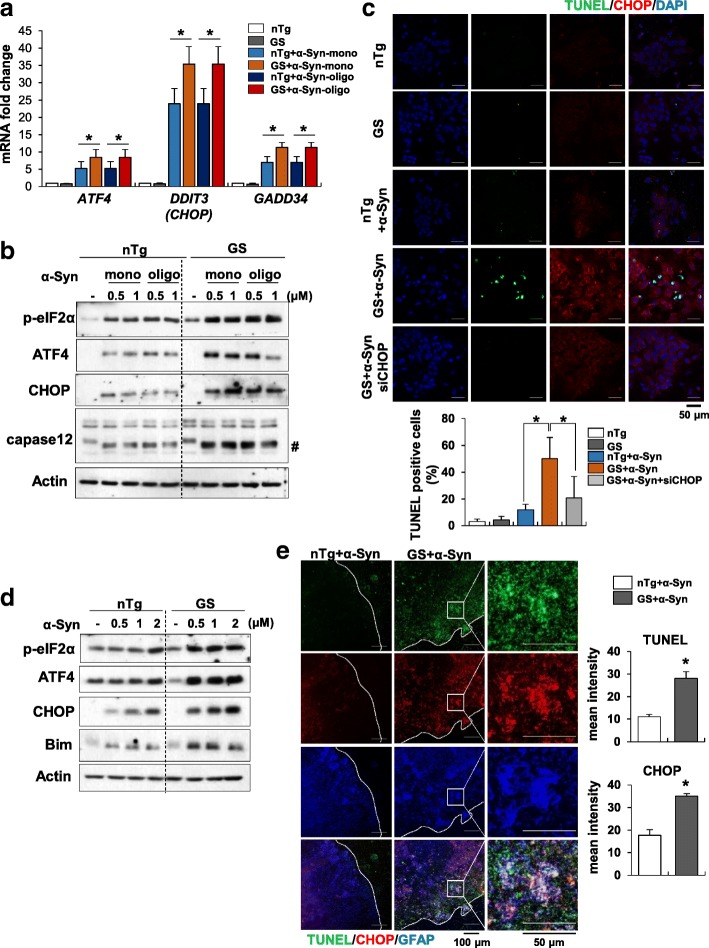
The LRRK2-GS mutant induces ER stress and apoptosis in astrocytes. **a**, **b** Astrocytes isolated from non-Tg (nTg) and LRRK2-GS (GS) mice were treated with monomeric (mono) or oligomeric (oligo) α-synuclein (α-Syn) for 48 h. Expression levels of the indicated mRNAs (**a**) and proteins (**b**) were analyzed by real-time qPCR and Western blotting, respectively. Crosshatching denotes a cleaved caspase-12 band. **c** Representative images (upper) and summary data (lower) showing TUNEL staining in astrocytes from non-Tg and LRRK2-GS mice treated with α-Syn for 72 h. **d**, **e** Organotypic slices from non-Tg and GS mice were treated with α-Syn (1 μM) for 48 h (**d**) or 72 h (**e**). Western blot showing expression levels of the indicated proteins (**d**) and representative images (left) and summary data (right) showing CHOP and TUNEL staining (**e**). All data are presented as means ± SD of three independent experiments (*p < 0.05)

Prolonged ER stress stimuli are associated with sustained activation of the UPR pathway and its switch to proapoptotic signaling. Of particular importance is the PERK pathway, which is involved inducing activating transcription factor 4 (ATF4), thereby controlling the expression of genes involved in cell death pathways, including CCAAT/enhancer-binding protein homologous protein (CHOP) [60]. Thus, we first assessed the severity of ER stress in astrocytes from LRRK2-G2019S mutant (LRRK2-GS-Tg) mice compared with those from non-transgenic (non-Tg) mice. To this end, we challenged non-Tg and LRRK2-GS astrocytes with monomeric or oligomeric α-synuclein for 24 h. Oligomeric α-synuclein incubated 2 days were used for experiments (Additional file 2: Figure S1a-b). We found that both types of α-synuclein induced apoptotic PERK-CHOP signaling in LRRK2-GS astrocytes, as evidenced by increases in mRNA and protein levels of the ER stress markers (Fig. 1 a-b). Since it is known that α-synuclein oligomers are more toxic conformation in PD, we choose oligomeric form of synuclein (0.5 μM) to induce ER stress in all experiments, though the monomeric and oligomeric α-synuclein have similar effect on ER stress response. To define the biological consequences of ER stress in LRRK2-GS astrocytes, we treated non-Tg and LRRK2-GS astrocytes with α-synuclein and quantified apoptotic cells using terminal deoxynucleotidyl transferase dUTP nick end labeling (TUNEL) assays. Apoptotic cells were frequent following treatment of LRRK2-GS astrocytes with α-synuclein, but were scarce among α-synuclein–treated non-Tg astrocytes (Fig. 1c). Small interfering RNA (siRNA)-mediated knockdown of CHOP markedly reduced the number of TUNEL-positive cells, suggesting that increased cell death in LRRK2-GS astrocytes is caused by severe ER stress. To further extend these findings, we tested whether similar events occurred under ex vivo conditions using brain slices obtained from non-Tg and LRRK2-GS mice. Compared with slices from non-Tg brains, LRRK2-GS brain slice cultures showed marked expression of ER stress-related proteins (Fig. 1d) and an increase in TUNEL/CHOP-positive astrocytes (Fig. 1e). Similar results were obtained following challenge with the ER-stress–inducing agent, tunicamycin (Additional file 2: Figure S1 c–f). Taken together, these results show that ER-stress–mediated cell death is significantly increased in LRRK2-GS astrocytes after α-synuclein treatment.

### G2019S-mutated LRRK2 is localized to the ER through disruption of 14–3-3 s binding

**Fig. 2:**
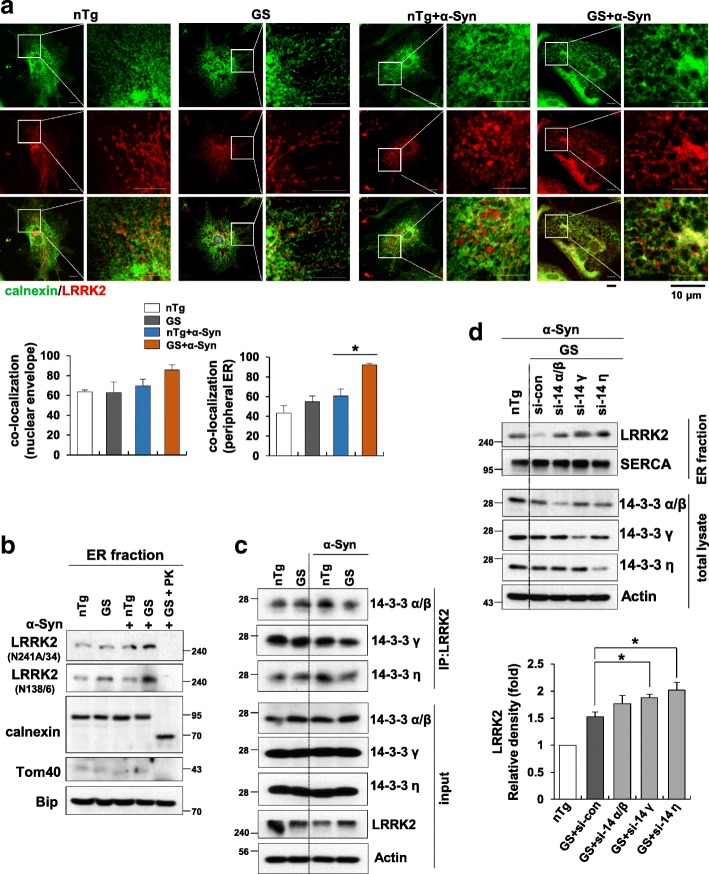
LRRK2-GS localizes to the ER membrane following dissociation from 14 to 3-3 s. **a** *Upper:* Confocal images of endogenous LRRK2 and calnexin in astrocytes isolated from non-Tg and LRRK2-GS mice. *Lower:* Statistical analysis of areas of overlap between LRRK2 and calnexin in nuclear envelope or peripheral ER regions. Images were analyzed using the Leica Application Suite and ImageJ software. Quantification was performed for 20 randomly selected cells from three independent experiments. **b** Western blot of the indicated proteins in ER-enriched fractions from non-Tg and LRRK2-GS astrocytes, incubated with or without proteinase K (PK). LRRK2 was detected using antibodies against the C- or N-terminus of LRRK2 (N241A/34 and N138/6, respectively). Calnexin, an integral membrane protein, Bip, an ER lumen protein, and Tom40, an import channel of the mitochondria outer membrane served as controls for the effect of PK. **c** Immunoprecipitation of LRRK2 in non-Tg and LRRK2-GS astrocytes, followed by Western blotting for the indicated proteins. **d** Western blot of the indicated proteins in ER fractions from non-Tg and LRRK2-GS astrocytes transfected with siRNAs targeting each 14–3-3 subtype. Band intensities were quantified densitometrically (lower). All graphs represent means ± SD of three independent experiments (*p < 0.05)

To study the underlying mechanism by which LRRK2-GS causes ER stress, we first determined whether LRRK2 is localized to the ER. To this end, we performed co-immunostaining of endogenous LRRK2 and calnexin, an ER membrane protein. Some overlap between the two proteins was found in α-synuclein treated non-Tg astrocytes, but most signals appeared as neighboring, but not colocalized, speckles scattered around the cytoplasm. In LRRK2-GS astrocytes, however, α-synuclein treatment caused greater ER targeting of LRRK2 to the ER in LRRK2-GS astrocytes than in non-Tg astrocytes (Fig. 2a and Additional file 2: Figure S2a). To confirm the precise location of LRRK2 within the ER, we performed a membrane proteinase “shaving” assay on LRRK2-GS and non-Tg astrocytes using an ER-enriched fraction. Briefly, ER-enriched fractions were first incubated with proteinase K (PK), after which the digestion products were analyzed for immunoreactivity with antibodies specific for the N- or C-terminal region of LRRK2 (N138/6 and N241A/34, respectively). Parallel immunoblots of the same lysates with anti-LRRK2 antibodies revealed a loss of immunoreactivity to both N- and C-terminal LRRK2 antibodies following PK treatment, indicating that LRRK2-GS was mainly bound to the cytosolic side of the ER membrane, rather than being localized within the ER (Fig. 2b and Additional file 2: Figure S2b). These results indicate greater localization of LRRK2 to the ER in LRRK2-GS astrocytes than in non-Tg astrocytes and suggest involvement of LRRK2-GS function in ER stress at the cytosolic side of the ER.

Previous reports have indicated that 14–3-3 proteins, a family of seven conserved adapter proteins, are involved in the localization of LRRK2 [9, 33]. 14–3-3 proteins bind to target proteins and modulate their activity, stability and/or subcellular localization; they also participate in many cellular functions, playing an important role in cell survival [8]. Recent reports have demonstrated that 14–3-3 s binding to LRRK2 is disrupted in some PD-related LRRK2 mutations, influencing its localization in the cell [21, 33]. Because we found that LRRK2-GS showed greater ER localization, we hypothesized that the interaction of 14–3-3 proteins with LRRK2 is altered in LRRK2-GS astrocytes. To test this, we performed co-immunoprecipitation of LRRK2 and various 14–3-3 isoforms using whole-cell lysates of LRRK2-GS and non-Tg astrocyte. We found that, although all six isoforms of 14–3-3 bound LRRK2, the γ and η isoforms showed diminished association with LRRK2 in LRRK2-GS astrocytes compared with non-Tg astrocytes (Fig. 2c and Additional file 2: Figure S2c). To verify the role of 14–3-3 s in LRRK2-GS localization, we measured LRRK2 protein levels in the ER-enriched fraction from astrocytes transfected with siRNA targeting individual 14–3-3 s subtypes. These experiments revealed that ER localization of LRRK2-GS was increased in cells in which the γ, or η isoform of 14–3-3 was knocked down (Fig. 2d). These results indicate that 14–3-3 proteins are involved in the localization of LRRK2 to ER, although the precise mechanism remains to be clarified.

### LRRK2-GS interacts with SERCA

**Fig. 3:**
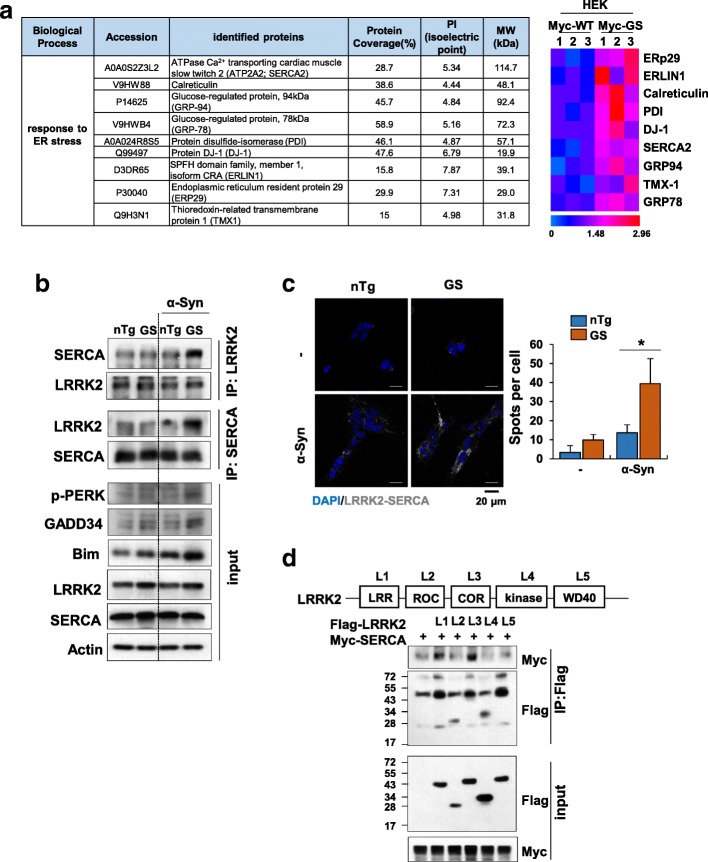
LRRK2-GS interacts with SERCA. **a** HEK293T cells were co-transfected with siRNA targeting the 3′-UTR region of LRRK2 and 3xMyc-tagged wild-type LRRK2 or G2019S-mutated LRRK2. After 48 h, cells were stimulated with α-synuclein (α-Syn) for 48 h and immunoprecipitated with antibody against Myc. LRRK2-interacting proteins were identified by MS analysis (Additional file 1: Table S1). This analysis revealed that LRRK2-GS–interacting proteins were enriched for Gene Ontology (GO) Biological Process terms associated with the ER stress response compared with LRRK2-WT–interacting proteins (> 1.5 fold, *p*-value < 0.05). Heatmap illustrating enriched LRRK2-GS–interacting proteins from three independent samples. The color bar represents fold change. **b**, **c** Astrocytes isolated from non-Tg and LRRK2-GS mice were treated with α-Syn for 48 h. Protein interactions were measured by immunoprecipitation with an antibody against LRRK2 or SERCA (**b**), and LRRK2–SERCA interactions were detected using the PLA method (**c**). PLA signals (in gray) were quantified, and displayed graphically as the average number of spots. Data are presented as means ± SD of three independent experiments (*p < 0.05). **d** HEK293T cells co-transfected with the indicated constructs were immunoprecipitated with an anti-Flag antibody, followed by immunoblot analysis with the indicated antibodies. Schematic diagram showing the structure of Flag-LRRK2

Next, in an effort to identify LRRK2-interacting proteins in the ER membrane, we performed label-free MS analyses of proteins co-immunoprecipitated with LRRK2 in α-synuclein–treated HEK293T cells transiently transfected with either Myc-tagged human wild-type LRRK2 (Myc-LRRK2-WT) or LRRK2-G2019S (Myc-LRRK2-GS). In total, 2831 proteins were identified, 232 of which showed increased interaction in LRRK2-GS–transfected cells compared with LRRK2-WT–transfected cells (Additional file 1: Table S1). A Cytoscape network analysis revealed that LRRK2-GS–interacting proteins could be categorized into five major groups according to Cellular Component: endoplasmic reticulum, proteasome complex, ribosomal subunit, ficolin-1 rich granule, and mitochondrial protein complex (Additional file 2: Figure S3a). A Biological Process gene ontology (GO) analysis further revealed that LRRK2-GS is involved in ER stress or UPR (Fig. 3a and Additional file 2: Figure S3b). To determine which of these ER-component proteins directly interacted with LRRK2-GS, we immunoprecipitated lysates of α-synuclein–treated non-Tg or LRRK2-GS astrocytes using antibodies against LRRK2 and then identified individual LRRK2-associated proteins in immunoprecipitates. Notable among these LRRK2-GS–interacting proteins was SERCA2/ATP2A2, an ER Ca^2+^ ATPase responsible for regulating Ca^2+^ transport across the ER membrane (Additional file 2: Figure S4a and Figure 3b). Enhanced LRRK2–SERCA2 interactions in LRRK2-GS astrocytes were further confirmed using proximity ligation assays (PLA) (Fig. 3c). Control experiments using the ER-stress–inducing agent tunicamycin instead of α-synuclein revealed prominent SERCA binding to LRRK2 (Additional file 2: Figure S4b).

To gain further molecular insights into LRRK2–SERCA interactions, we transfected HEK293T cells with full-length Myc-tagged SERCA and Flag-tagged, truncated mutants of LRRK2, and analyzed SERCA–LRRK2 binding by immunoprecipitation and Western blotting. These analyses showed that leucine-rich repeat (LRR) and carboxy-terminal of Roc (COR) domains of LRRK2 interacted with SERCA (Fig. 3d). These results are consistent with previous reports that LRR and COR domains participate in protein-protein and intramolecular interactions [14, 51], and collectively confirm that LRRK2 and SERCA directly bind each other through LRRK2 LRR and COR domains.

Because we found that 14–3-3 s are involved in ER localization of LRRK2-GS (Fig. 2), we tested whether 14–3-3 s affects LRRK2-GS–SERCA interactions. As expected, LRRK2-GS–SERCA interactions were increased by siRNA-mediated knockdown of γ and η forms of 14–3-3 s compared with that observed in the si-control–transfected LRRK2-GS group (Additional file 2: Figure S4 c-d). These results indicate that LRRK2-GS freed from 14 to 3-3 s localizes to the ER and interacts with SERCA.

### LRRK2-GS promotes ER Ca^2+^ depletion by maintaining SERCA–PLN interactions

**Fig. 4:**
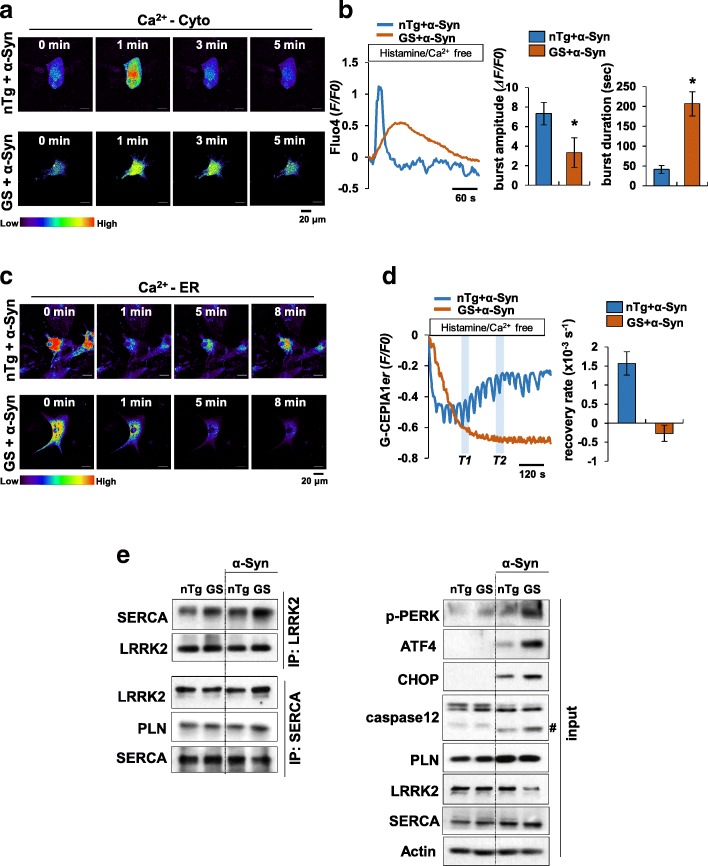
LRRK2-GS induces ER Ca^2+^ depletion by maintaining SERCA–PLN interactions. **a**, **b** Representative time-lapse images of cytosolic Ca^2+^ dynamics in astrocytes isolated from non-Tg and LRRK2-GS mice. Astrocytes were treated with α-synuclein (α-Syn) for 24 h, after which cytosolic Ca^2+^ signals in response to histamine (500 μM) were visualized by fluorescence microscopy in cells loaded with Fluo4-AM. Fluorescence intensity is displayed in pseudo-colors (bottom: scale palette) (a). Representative Ca^2+^ transients were plotted (b; left) and the average amplitude and duration (b; right) of Ca^2+^ spark were quantified. **c**, **d** Representative images of ER Ca^2+^ dynamics in astrocytes expressing G-CEPIA1*er*. Astrocytes were treated with α-Syn, and then Ca^2+^ signals were visualized by fluorescence microscopy. Changes in fluorescence intensities are indicated by pseudo-colors (c). Plots of representative fluorescence dynamics (d; left), and the slope of a linear fit to G-CEPIA1*er* fluorescence changes during the interval *T1* to *T2* in left panel of D was obtained; the indicated differences are shown (d, right). **e** Astrocytes isolated from non-Tg and LRRK2-GS mice were treated with α-Syn and immunoprecipitated with an antibody against LRRK2 or SERCA, followed by immunoblot analysis with the indicated antibodies. Crosshatching denotes a cleaved caspase-12 band. All graphs represent means ± SD of three independent experiments (*p < 0.05)

Because LRRK2-GS is co-localized and physically associated with SERCA, we examined whether Ca^2+^ flux is altered in LRRK2-GS astrocytes using the fluorescent cytosolic Ca^2+^ indicator dye, Fluo4-AM. To this end, we pretreated LRRK2-GS and non-Tg astrocytes with α-synuclein for 24 h, loaded cells with Fluo4-AM. Cells were then stimulated with histamine, which generates cytosolic Ca^2+^ oscillations by inducing Ca^2+^ release from the ER via inositol 1,4,5-trisphosphate receptors (IP_3_Rs). Following stimulation with histamine, the peak amplitude of Ca^2+^ bursts was decreased in LRRK2-GS astrocytes compared with that in non-Tg astrocytes, reflecting ER Ca^2+^ store depletion by α-synuclein treatment. Moreover, the duration of Ca^2+^ bursts was longer owing to malfunction of SERCA in LRRK2-GS astrocytes (Fig. 4 a-b; Online Resource 1 and 2). To further confirm this, we determined ER Ca^2+^ dynamics using G-CEPIA1*er* (calcium-measuring organelle-entrapped protein indicator 1 in the ER), a genetically encoded, GFP-tagged ER Ca^2+^ indicator. In non-Tg astrocytes, stimulation of astrocytes with histamine induced depletion of ER Ca^2+^ followed by recovery toward the pre-stimulation level. In LRRK2-GS astrocytes, however, fluorescence intensity was reduced and ER Ca^2+^ refilling was blocked. Measurements of ER Ca^2+^ recovery rate showed that ER Ca^2+^ was scarcely reloaded in LRRK2-GS astrocytes, but was readily reloaded in non-Tg astrocytes (Fig. 4 c-d; Online Resource 3 and 4). These results suggest that LRRK2–SERCA interactions in LRRK2-GS astrocytes cause malfunction of SERCA, resulting in ER Ca^2+^ depletion, and possibly increased ER stress-induced cell death.

SERCA activity is mediated in various ways. A recent study reported that small-conductance Ca^2+^-activated potassium (SK) channels are present in the ER of cardiomyocytes and neurons, where they regulate ER Ca^2+^ uptake and protect against ER-stress–induced cell death [44]. We therefore investigated the effects of SK channel activation in our model of ER stress-induced cell death. For this, we applied cyclohexyl-[2-(3,5-dimethyl-pyrazol-1-yl)-6-methyl-pyrimidin-4-yl]-amine (CyPPA), a positive modulator of SK channels that is selective for SK2 and SK3 subtypes. However, application of CyPPA did not protect against α-synuclein–induced ER stress in LRRK2-GS astrocytes, indicating that SK2 and SK3 channels are not involved in LRRK2-GS–mediated SERCA inactivation (Additional file 2: Figure S5a). It has also been reported that SERCA activity is regulated by the ER protein chaperones, calreticulin (CRT), calnexin (CLNX), and ER protein 57 (ERp57). Although the precise molecular mechanism is not completely known, CRT and CLNX can regulate SERCA directly or possibly indirectly by recruiting other enzymes, such as ERp57 [46, 62]. To determine whether these chaperones are involved in SERCA inactivation, we performed co-immunoprecipitation assays in non-Tg and LRRK2-GS astrocytes using antibodies against SERCA and individual chaperone proteins. However, we found no significant difference in SERCA-chaperone interactions between non-Tg and LRRK2-GS astrocytes. Some studies have suggested that redox-sensitive selenoprotein N (SEPN1) and thioredoxin-related transmembrane protein 1 (TMX1) bind to loop 4 cysteines in SERCA and regulate ER Ca^2+^ uptake activity [27, 42]. But we found that these potential regulators also showed no difference in their interactions with SERCA between non-Tg and LRRK2-GS astrocytes (Additional file 2: Figure S5b). Finally, we explored the involvement of the small transmembrane protein PLN, which decreases ER Ca^2+^ uptake through association with SERCA. Protein kinase A (PKA)-mediated phosphorylation of PLN causes PLN dissociation from SERCA, permitting near-maximal Ca^2+^-ATPase activity and increased ER Ca^2+^ loading in cardiac and skeletal muscle [25]. Although it is known that PLN is expressed almost exclusively in muscle cells, we confirmed expression of PLN protein in astrocytes, but not neurons (Additional file 2: Figure S5c). We then co-immunoprecipitated SERCA and PLN from lysates of non-Tg and LRRK2-GS astrocytes. These analyses showed that SERCA–PLN interactions were increased in LRRK2-GS astrocytes compared with non-Tg astrocytes (Fig. 4e), suggesting that LRRK2-GS–SERCA interactions inhibit dissociation of PLN from SERCA. Taken together, these findings suggest that LRRK2-GS mediates SERCA inactivation through maintenance of PLN–SERCA interactions, thereby leading to continued ER Ca^2+^ depletion.

### LRRK2-GS increases ER-mitochondrial contact and leads to mitochondrial Ca^2+^ overload and dysfunction

**Fig. 5:**
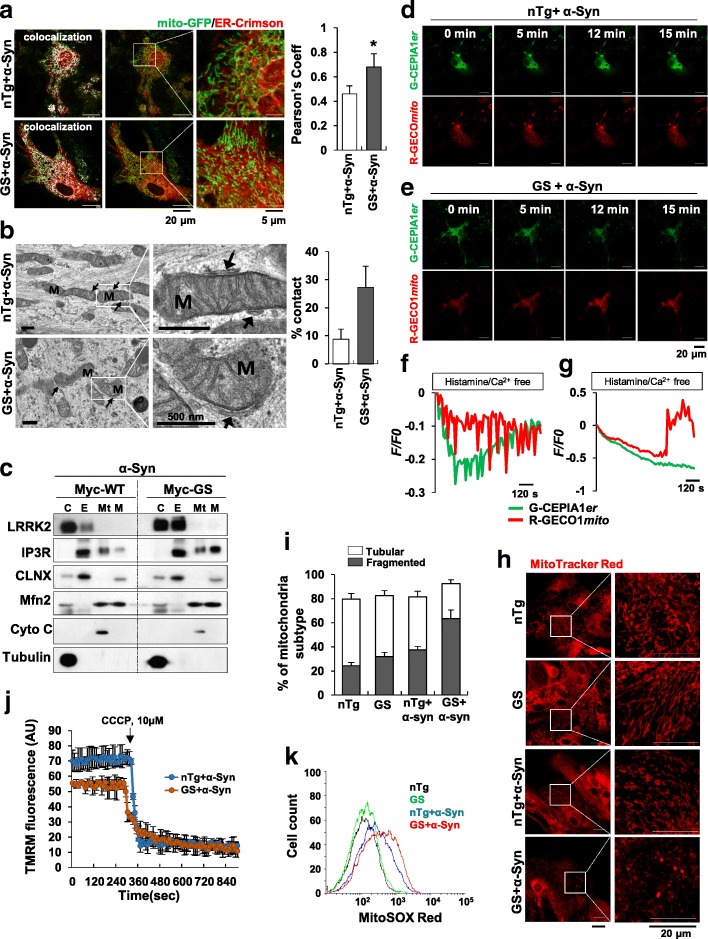
ER stress and Ca^2+^ depletion in LRRK2-GS astrocytes induce mitochondrial dysfunction. **a** *Left:* Confocal images of astrocytes isolated from non-Tg or LRRK2-GS mice co-expressing mitochondria-targeted GFP (mito-GFP) and ER-targeted E2-Crimson (ER-Crimson). *Right:* Statistical analysis of the area of overlap (Pearson’s coefficient) between mito-GFP and ER-Crimson. **b** Representative electron micrographs of ER–mitochondria associations in non-Tg and LRRK2-GS astrocytes; arrows indicate the ER; M, mitochondria. Bar chart shows the percentage of the mitochondrial surface in close apposition to the ER. Data are presented as means ± SD (error bars). *p < 0.05; *n* = 50 mitochondria from 10 cells. **c** Western blot analysis of the indicated proteins in subcellular fractions from HEK293T cells expressing Myc-LRRK2-WT or Myc-LRRK2-GS. C, cytosol; E, ER; Mt., pure mitochondria; M, MAM. **d-g** Representative time-lapse fluorescence images of G-CEPIA1*er*-and R-GECO1*mito*-expressing non-Tg (**d**) and LRRK2-GS (**e**) astrocytes, and plots of Ca^2+^ signals in the ER and mitochondria of non-Tg (f) and LRRK2-GS (**g**). Astrocytes were co-transfected with G-CEPIA1*er* and R-GECO1*mito*, and then treated with α-synuclein (α-Syn). After 24 h, cells were stimulated with histamine and imaged by fluorescence microscopy. **h** Morphology of mitochondria in non-Tg and LRRK2-GS astrocytes stained with MitoTracker Red CMXRos (250 nM). **i** Quantification of tubular and fragmented mitochondria using a semi-automated system. Images from three independent areas containing 200–300 mitochondria from 5 cells were analyzed from each group. **j** Mitochondrial membrane potential in astrocytes isolated from non-Tg and LRRK2-GS mice. AU, arbitrary units. **k** Representative ROS production measured in cells loaded with MitoSOX (5 μM). All graphs represent means ± SD of three independent experiments (*p < 0.05)

In addition to distinct and independent roles of the ER and mitochondria, accumulating recent evidence suggests that the two organelles physically and functionally interact at sites designated mitochondria-associated ER membranes (MAMs) [36]. MAMs have a key regulatory role in various cellular functions, including the transfer of Ca^2+^ and lipids from the ER to mitochondria, regulation of mitochondrial dynamics, and formation of signaling scaffolding [23]. These ER contact sites are enriched in specific proteins, such as IP_3_R Ca^2+^ channels; the mitochondrial-ER tethering proteins, mitofusin-2 (Mfn2) and phosphofurin acidic cluster sorting protein 2 (PACS2); and chaperones, such as sigma 1 receptor (Sig1R) and CLNX. Accordingly, dysfunctions in one organelle can affect the other, leading to various pathologies, including neurodegenerative diseases [36, 48]. Under conditions of cell stress, particularly ER stress, the extent of MAMs and magnitude of Ca^2+^ flux from the ER to mitochondria increases, producing considerable amounts of reactive oxygen species (ROS) and inducing opening of the mitochondrial permeability transition pore (mPTP), promoting proapoptotic signaling [45]. Because we found that interactions of LRRK2-GS with SERCA disrupt Ca^2+^ homeostasis and increase ER stress, we sought to verify ER-to-mitochondrial function in non-Tg and LRRK2-GS astrocytes. First, to investigate ER-mitochondrial interactions, we co-expressed mitochondria-targeted GFP (mito-GFP) and ER-targeted E2-Crimson (ER-Crimson) in non-Tg and LRRK2-GS astrocytes and imaged cells by fluorescence microscopy. The proportion of ER and mitochondria colocalization in LRRK2-GS astrocytes was greater than that in non-Tg astrocytes (Fig. 5a). Transmission electron microscopy (TEM) images further revealed that the proportion of the ER in close contact with mitochondria was higher in LRRK2-GS astrocytes than non-Tg astrocytes (Fig. 5b). To confirm these results, we performed Percoll-based subcellular fractionation of HEK293T cells transfected with Myc-LRRK2-WT or Myc-LRRK2-GS, and collected fractions enriched for mitochondria, the ER, or MAM. IP_3_R and CLNX were preferentially found in ER fractions, whereas cytochrome C and Mfn2 were enriched in mitochondrial fractions, results in line with previous reports [43]. Interestingly, the levels of IP_3_R, CLNX and Mfn2 in MAM fractions were higher in Myc-LRRK2-GS–transfected cells than in Myc-LRRK2-WT–transfected cells (Fig. 5c), suggesting a possible redistribution of these proteins from the ER or mitochondrial membrane to MAMs. Taken together, these results indicate that ER-mitochondria contact sites are increased in LRRK2-GS astrocytes.

The fact that LRRK2-GS disrupts Ca^2+^ homeostasis in the ER by interacting with SERCA (Figs. 3 and 4), taken together with the increase in Ca^2+^ regulatory proteins in MAMs of LRRK2-GS astrocytes, led us to postulate that Ca^2+^ flux from the ER to mitochondria is increased in LRRK2-GS astrocytes, with deleterious effect on mitochondrial functions. To address this possibility, we assessed ER and mitochondria Ca^2+^ dynamics by expressing G-CEPIA1*er* or R-GECO1*mito*, indicators of ER and mitochondria Ca^2+^, respectively, in non-Tg and LRRK2-GS astrocytes. In non-Tg astrocytes, histamine stimulation induced depletion of ER Ca^2+^, with subsequent recovery of ER Ca^2+^ to pre-stimulation levels. Despite these changes in ER Ca^2+^, mitochondrial Ca^2+^ levels were largely unchanged (Fig. 5 d a-f; Online Resource 5). In contrast to non-Tg astrocytes, LRRK2-GS astrocytes exhibited ER Ca^2+^ depletion and a burst in elevated mitochondrial Ca^2+^ uptake (Fig. 5 e-g; Online Resource 6), suggesting that Ca^2+^ flux from the ER to mitochondria was increased in LRRK2-GS astrocytes. Next, we determine whether mitochondrial Ca^2+^ overload was accompanied by mitochondrial dysfunction. First, we examined morphological changes in mitochondria in non-Tg and LRRK2-GS astrocytes. To this end, we stained cells with MitoTracker and assigned mitochondria to one of two categories: tubular or fragmented. The number of LRRK2-GS astrocytes containing fragmented mitochondria was increased compared with non-Tg astrocytes (Fig. 5 h-i). We also found that mitochondrial membrane potential was lower (Fig. 5j) and mitochondrial ROS levels were higher (Fig. 5k) in LRRK2-GS astrocytes, as measured by tetramethyl rhodamine methyl ester (TMRM) and MitoSOX fluorescence, respectively. These data, taken together with the altered mitochondrial morphology observed in LRRK2-GS astrocytes, demonstrate that high Ca^2+^ flux from the ER to mitochondria, caused by LRRK2-GS–mediated SERCA inactivation, is associated with mitochondrial dysfunction.

### LRRK2 kinase activity is not required for LRRK2 localization to the ER

It is well known that the LRRK2-GS mutation in the kinase domain leads to increase in catalytic activity, a change that underlies the neurotoxic effect of this mutant [12, 55]. Moreover, treatment with LRRK2 kinase inhibitors mitigates toxicity and neurodegeneration both in vitro and in vivo [5, 20]. However, there are conflicting reports regarding the role of LRRK2 kinase activity in ER dysfunction and cell death. Yuan and colleagues reported that LRRK2-GS causes cell death through chronic p38 activation in an LRRK2 kinase-dependent manner [65], whereas another study suggested that LRRK2 kinase activity may not regulate the interaction of Sec16A with LRRK2, which leads to ER-Golgi transport dysfunction [3]. Our fractionation experiments showed that α-synuclein–induced ER translocation of LRRK2 was not suppressed by treatment with the LRRK2 kinase inhibitors, IN-1 or PF-06447475 (Additional file 2: Figure S6a), suggesting that this translocation is independent of LRRK2 kinase activity. In line with ER fractionation assays, immunoprecipitation experiments further showed that LRRK2-SERCA interactions were not significantly changed by treatment with either kinase inhibitor (Additional file 2: Figure S6b, upper panel). Contrary to our expectation, however, expression of ER stress and cell death marker proteins were suppressed by both inhibitors (Additional file 2: Figure S6b, lower panel; Additional file 2: Figure S6c). These findings suggest that LRRK2-GS causes ER stress through a mechanism that involves LRRK2 kinase activity-independent SERCA inactivation, or another pathway that is affected by enhanced LRRK2 kinase activity.

### LRRK2-GS astrocytes cause death of cortical neurons

**Fig. 6:**
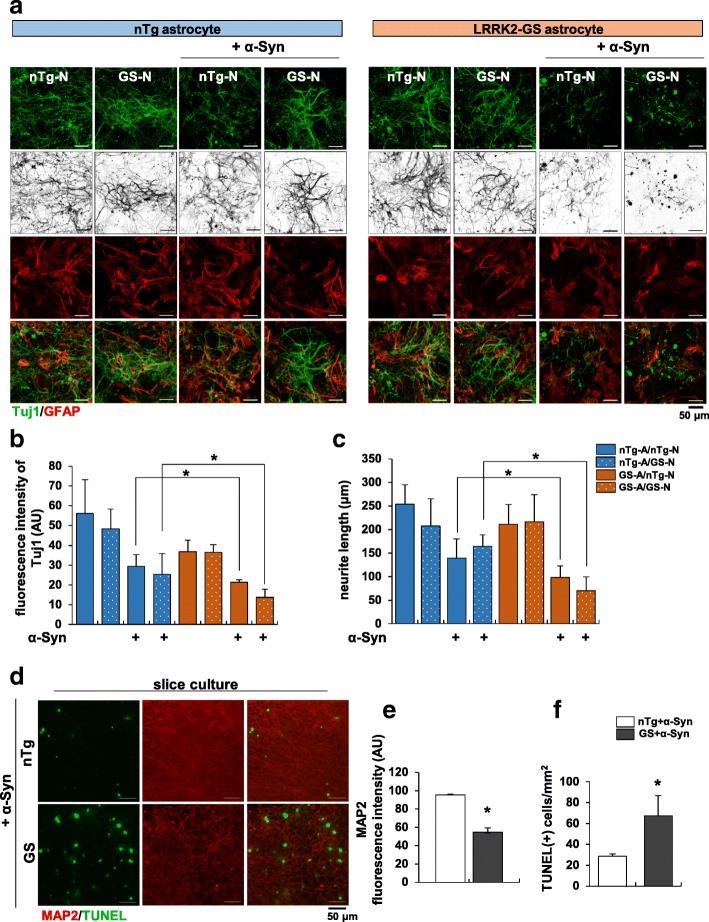
Astrocytes with ER stress-induced abnormalities affect neuronal survival. **a** Neurons isolated from non-Tg or LRRK2-GS mice were layered on top of non-Tg or LRRK2-GS astrocytes for 7–10 days in vitro, allowing contact between neurons and astrocytes. Representative double-immunofluorescence staining of direct co-cultures with anti-GFAP and Tuj1 antibodies; cells were incubated in the absence or presence of α-synuclein (α-Syn). **b**, **c** Summary data showing the fluorescence intensities of Tuj1 per cell (*n* = 25) (**b**) and average length of neurites per cell (*n* = 30 cells) (**c**). **d-f** Organotypic slices from non-Tg and LRRK2-GS mice were treated with α-Syn for 72 h. Representative images of TUNEL staining (**d**), and graphs showing fluorescence intensities of MAP2 per each independent area (*n* = 5) (**e**) and TUNEL-positive cells per mm^2^ (*n* = 6) (**f**). All data are presented as means ± SD of three independent experiments (*p < 0.05)

Because astrocytes secrete a range of factors, including neurotrophic factors, growth factors and cytokines that stimulate remyelination by promoting neuronal survival, neurite outgrowth and neurogenesis, astrocyte dysfunction might drive neuronal disease [17]. In this context, death of LRRK2-GS astrocytes owing to ER stress could aggravate neuronal damage and death. To address whether LRRK2-GS astrocytes are involved in the dysfunction and loss of cortical neurons, we used a coculture system in which non-Tg or LRRK2-GS cortical neurons were directly plated onto a feeder layer of non-Tg or LRRK2-GS astrocytes (Additional file 2: Figure S7a). In non-Tg as well as LRRK2-GS neuron plated on LRRK2-GS astrocytes, α-synuclein treatment decreased expression of the neuron-specific class III beta-tubulin (Tuj1) and neurite length, supporting the concept that ER stress-induced death of LRRK2-GS astrocytes aggravates neuronal damage (Fig. 6 a–c). In a complementary approach, we collected astrocyte-conditioned medium (ACM) from non-Tg or LRRK2-GS astrocytes and applied it to primary cortical neurons (Additional file 2: Figure S7b). We found that treatment of neurons with ACM from LRRK2-GS astrocytes decreased neurite length and the percentage of cells positive for microtubule-associated protein 2 (MAP2), a marker for differentiated neurons, compared with treatment with ACM from non-Tg astrocytes (Additional file 2: Figure S7 c-d). Moreover, the percentage of MAP2+ neurons was sharply reduced in brain slices from LRRK2-GS mice, in association with an increase in TUNEL-positive cells, compared with non-Tg brain slices (Fig. 6 d-f). Previous studies have shown that a transgenic mouse model in which human LRRK2-GS is overexpressed exhibits a modest loss of dopaminergic neurons accompanied by accumulation of autophagosomes, mitochondrial alterations and ER stress, revealing direct toxic effect of LRRK2-GS on neurons [41, 65]. In our study, however, we found no difference in SERCA–LRRK2 interactions, expression of ER stress proteins, or cell death between non-Tg and GS neurons. Moreover, PLN is not expressed in neurons (Additional file 2: Figure S7e), as previously reported [40]. These results suggest that the functions of LRRK2-GS in neurons and astrocytes differ with respect to ER stress. Specifically, neuronal damage and death in LRRK2-GS mice reflect the effects of abnormal astrocytes, rather than a direct effect of LRRK2-GS on neurons.

### LRRK2-GS is also involved in enhanced ER stress in other cell types

**Fig. 7:**
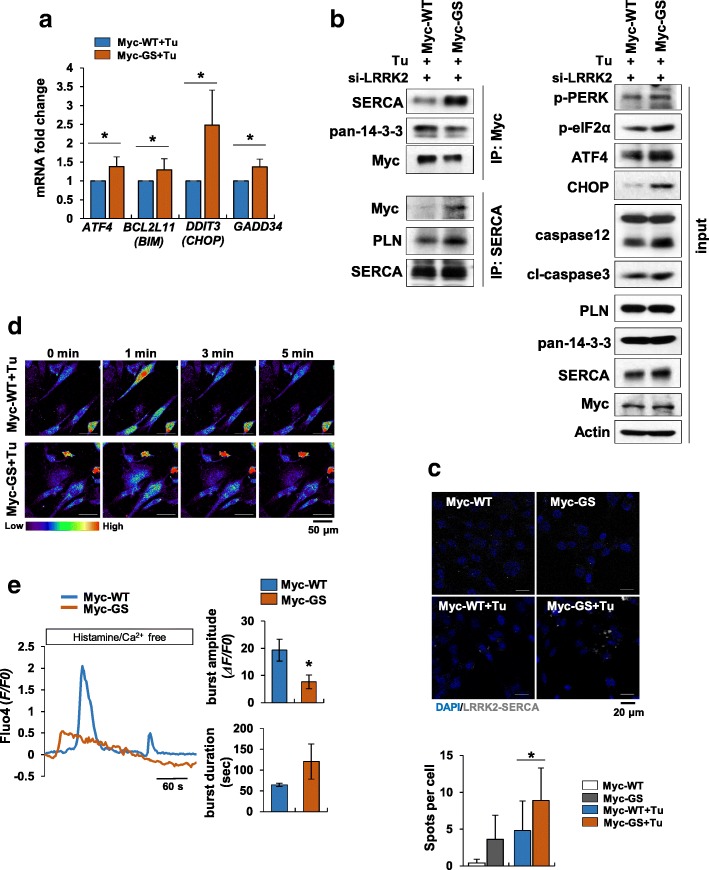
ER stress mediated by LRRK2-GS is not specific to astrocytes. C2 myoblasts expressing Myc-LRRK2-WT or Myc-LRRK2-GS were treated with tunicamycin (0.1 μg/ml) for 24–48 h. **a** Levels of the indicated mRNAs were analyzed by real-time qPCR. **b**, **c** Protein interactions were detected by immunoprecipitation with an antibody against Myc or SERCA followed by Western blotting (**b**), and LRRK2–SERCA interactions were detected using the PLA method (**c**). **d**, **e** Representative time-lapse images of cytosolic Ca^2+^ dynamics in response to histamine, visualized by fluorescence microscopy in C2 myoblasts loaded with Fluo4-AM. Fluorescence intensity, displayed in pseudo-colors (bottom: scale palette) (**d**), plots of representative Ca^2+^ transients (**e**, left), and quantification of average amplitude and duration of Ca^2+^ sparks (**e**, right). All data are presented as means ± SD of three independent experiments (*p < 0.05)

The role of SERCA in refilling intracellular Ca^2+^ stores is pivotal for maintaining intracellular Ca^2+^ homeostasis. Accordingly, disturbed SERCA activity is involved in a variety of diseases, including heart failure, diabetes, cancer, and Alzheimer’s disease [2]. The importance of the SERCA pump in cardiac and skeletal muscle function, in particular, has been extensively studied. [1]. Because we showed that LRRK2-GS promotes Ca^2+^ dyshomeostasis by maintaining SERCA–PLN interactions, we explored whether the effect of LRRK2-GS on SERCA inactivation was general or astrocyte specific. To this end, we expressed Myc-LRRK2-WT or Myc-LRRK2-GS in C2 myoblasts, a skeletal muscle cell line, and treated them with tunicamycin. We found that pro-apoptotic PERK-CHOP signaling was increased in LRRK2-GS–expressing C2 cells, as determined by an assessment of mRNA and protein expression of the ER stress markers by qRT-PCR and Western blotting, respectively (Fig. 7 a-b). Moreover, SERCA–LRRK2 interactions were increased in LRRK2-GS–expressing C2 cells compared with LRRK2-WT–expressing cells (Fig. 7b). Enhanced LRRK2–SERCA interactions in LRRK2-GS–expressing C2 cells were further confirmed using the PLA method (Fig. 7c). To determine whether the increase in LRRK2–SERCA interactions in LRRK2-GS–expressing C2 cells subsequently affects ER stress responses, we determined whether Ca^2+^ flux was altered in this alternative cell type using Fluo4-AM. Peak Ca^2+^ burst amplitude and duration were decreased in LRRK2-GS–expressing C2 cells compared with LRRK-WT–expressing cells, indicating that SERCA does not function properly in LRRK2-GS–mutated muscle cells (Fig. 7 d-e; Online Resource 7 and 8). Although only a single additional cell type was examined in this study, these results suggest that the effects of LRRK2-GS on ER stress through regulation of SERCA activity is a general phenomenon in certain cell types in which SERCA activity is a prominent feature.

## Discussion

In the current study, we demonstrated that LRRK2-GS acts through SERCA inactivation to trigger ER stress in α-synuclein treated-brain astrocytes, leading to cell death. SERCA is inactivated by its direct interaction with LRRK2-GS, which is translocated to the ER after its release from 14 to 3-3 s. ER-translocated LRRK2-GS persistently maintains the SERCA–PLN complex, thereby disrupting SERCA function and causing ER Ca^2+^ depletion. Furthermore, LRRK2-GS induced the formation of MAMs and caused Ca^2+^ overload in mitochondria, leading to mitochondrial dysfunction. Intriguingly, this phenomenon was observed in skeletal muscle cells, another cell type in which SERCA function is prominent.

In most cell types, the ER is the largest intracellular organelle and extends throughout the cytosol. In addition to its role in storing, modifying and transporting newly synthesized proteins, the ER is a high-capacity reservoir for intracellular Ca^2+^. SERCAs are responsible for maintenance of the micro- to millimolar Ca^2+^ ion concentrations within the ER of eukaryotic cells. Because ER-resident chaperones like CRT, BiP and GRP94 require high Ca^2+^ concentrations for their protein-folding activity, the failure of ER Ca^2+^ homeostasis resulting from SERCA malfunction brings about an imbalance between the capacity of the protein processing machinery and the amount of unfolded proteins accumulated in the ER, leading to ER-stress–mediated apoptosis [24]. Moreover, inappropriate Ca^2+^ flux from the ER to mitochondria through MAMs impairs several mitochondrial processes. Basal Ca^2+^ oscillations drive the mitochondrial metabolism necessary for the production of ATP and mitochondrial substrates used in anabolic processes. In contrast, mitochondrial Ca^2+^ overload can cause cell death [19]. Specifically, this latter study showed that inhibition of SERCA with thapsigargin efficiently killed BAX/BAK^−/−^ mouse embryo fibroblasts (MEFs) by inducing mitochondrial Ca^2+^ overload and opening of the mPTP [15]. Recent studies have reported that dysregulation of Ca^2+^ flux through inactivation of SERCA is involved in several neurological diseases, such as Sandhoff disease and Niemann-Pick A disease [38]. Using a neuropathic pain model, Gemes and colleagues found that spinal nerve ligation causes a loss of SERCA that results in depletion of ER Ca^2+^, and suggested that this may trigger a UPR [11]. These observations highlight the importance of seeking approaches for restoring appropriate regulation of SERCA function in the treatment of various diseases. In the present study, we showed that LRRK2-GS directly binds and regulates SERCA. This LRRK2-GS–SERCA interaction maintains SERCA–PLN interactions, leading to malfunction of SERCA in the ER (Figs. 3 and 4). PLN is profusely expressed in muscle cells and its role in these cells is well known, but the roles and expression patterns of PLN in the brain are largely unknown. One study reported that PLN is not expressed in neurons [40], whereas another suggested that Mn^2+^ induced the expression of PLN transcripts in astrocytes [54]. In the current study, we detected PLN expression in both non-Tg and LRRK2-GS astrocytes, but not in neurons (Additional file 2: Figure S5), suggesting that the effect of LRRK2-GS on SERCA may originate from differential expression of PLN. Results obtained in C2 myoblasts further support the idea that this LRRK2-GS mechanism of action is applicable to other cells that express PLN (Fig. 7). Further studies are needed to elucidate whether this is a global or context-specific phenomenon.

The mechanisms that lead to ER dysfunction in PD remain incompletely understood. Moreover, the link between ER dysfunction and PD-related genes, specifically LRRK2, is just starting to be uncovered. Mutated LRRK2 is indirectly involved in ER dysfunction in various ways. First, inhibition of autophagy by mutant LRRK2 might trigger ER dysfunction, given that impairment of autophagy, particularly chaperone-mediated autophagy (CMA), is a reported feature of mutant LRRK2 animal models [35, 61]. Accordingly, impaired autophagy could be a contributing factor to increased α-synuclein aggregation, which initiates aggregated α-synuclein-dependent ER dysfunction, thereby exacerbating the increase in autophagic cargo and further inhibiting CMA. Consequently, LRRK2 mutations further aggravate ER dysfunction. Secondly, perturbation of endosomal trafficking by mutant LRRK2 imposes a burden on ER. LRRK2 has been shown to interact with several Rab family proteins, which are important regulators of intracellular vesicular trafficking. Mutant LRRK2 phosphorylates Rab, probably leading to altered interactions with downstream effectors of Rab, as well as perturbations in endosome-to-lysosome trafficking [26, 59]. That means α-synuclein failed to be degraded by lysosomes in mutant LRRK2, adding further pressure on the ER that ultimately leads to ER dysfunction. Lastly, mitochondrial dysfunction caused by mutant LRRK2 impairs physiological functions of the ER. Mutant LRRK2 induces the expression of Bim, a pro-apoptotic BH3-only family member, by phosphorylating the transcription factor FoxO [16]. Many of these BH3-only proteins affect ER Ca^2+^ homeostasis by binding to the IP_3_R and/or changing its phosphorylation status, thereby altering the Ca^2+^-flux properties of the channel [47]. Moreover, PD-linked LRRK2 mutations increase mitochondrial Ca^2+^ uptake in cortical neurons in association with increased expression of the mitochondrial Ca^2+^ uniporter (MCU) [63]. As shown by our MS/MS analysis in the current study, LRRK2-GS also interacts with proteins involved in mitochondria membrane organization and mitochondrial transport, suggesting that LRRK2 may contribute to mitochondria function in astrocytes (Additional file 1: Table S1 Additional file 2: Figure S3). Accordingly, the effect of mutant LRRK2 on mitochondria may induce de novo ER stress or aggravate existing ER dysfunction.

In this study, we demonstrated the molecular mechanism by which G2019S-mutated LRRK2 induces ER stress and subsequent cell death. By incorporating previously unidentified LRRK2-interacting components, this study fills an important gap in our understanding of the relationship between LRRK2-GS and PD pathogenesis. Specifically, our study suggests a plausible model that links LRRK2-GS to pathophysiological ER stress in PD. In addition, novel aspects of this work related to mutated LRRK2 in muscle cells will help shed light on how mutated LRRK2 leads to abnormal phenotypes in peripheral tissues. A better understanding of LRRK2 biology in the context of ER stress could impact ongoing efforts to establish the action mechanism of LRRK2 in both PD and peripheral organs.

## Conclusion

In conclusion, we found that LRRK2-GS acts through SERCA inactivation to trigger ER stress in α-synuclein treated-brain astrocytes, leading to cell death. The data showed that SERCA is inactivated by its direct interaction with LRRK2-GS, which is translocated to the ER after its release from 14-3-3s. ER-translocated LRRK2-GS persistently maintains the SERCA–PLN complex, thereby disrupting SERCA function and causing ER Ca^2+^ depletion. Also, LRRK2-GS induced the formation of MAMs and caused Ca^2+^ overload in mitochondria, leading to mitochondrial dysfunction. Overall, these data shed light on potential mechanisms underlying LRRK2-GS’s actions in ER stress that renders severe neurological diseases such as PD.

## Additional file

Additional file 1: **Table S1.** List of LRRK2 interacting proteins, Related Figure 3. (XLSX 41 kb)

Additional file 2: **Figure S1.** The LRRK2-GS mutant induces ER stress and apoptosis in tunicamycin-treated astrocytes. **Figure S2.** LRRK2-GS is localized to the ER membrane. **Figure S3.** Analysis of GS-LRRK2–interacting proteins. **Figure S4.** Identification of GS-LRRK2–interacting proteins. **Figure S5.** LRRK2-GS promotes SERCA–PLN complex formation. **Figure S6.** LRRK2 kinase activity does not affect LRRK2 localization. **Figure S7.** LRRK2-GS astrocytes affect neuronal survival. **Table S2.** List of siRNA oligonucleotides. **Table S3.** List of primers used for qPCR. **Table S4.** List of primary antibodies used in this work. (PDF 5703 kb)
