# Supplementary material for: Parkinson’s disease-associated LRRK2-G2019S mutant acts through regulation of SERCA activity to control ER stress in astrocytes
Source: Acta Neuropathol Commun. 2019 May 2;7:68. doi: 10.1186/s40478-019-0716-4 (PMC6498585; doi:10.1186/s40478-019-0716-4)
Supplement: Supplementary file 2 — Figure S1. The LRRK2-GS mutant induces ER stress and apoptosis in tunicamycin-treated astrocytes. Figure S2. LRRK2-GS is localized to the ER membrane. Figure S3. Analysis of GS-LRRK2–interacting proteins. Figure S4. Identification of GS-LRRK2–interacting proteins. Figure S5. LRRK2-GS promotes SERCA–PLN complex formation. Figure S6. LRRK2 kinase activity does not affect LRRK2 localization. Figure S7. LRRK2-GS astrocytes affect neuronal survival. Table S2. List of siRNA oligonucleotides. Table S3. List of primers used for qPCR. Table S4. List of primary antibodies used in this work. (PDF 5703 kb) [file 40478_2019_716_MOESM2_ESM.pdf]

Parkinson's disease-associated LRRK2-G2019S mutant acts through regulation of SERCA activity to control ER stress in astrocytes

Jee Hoon Lee, Ji-hye Han, Hyunmi Kim, Sang Myun Park, Eun-hye Joe and Ilo Jou*

Department of Pharmacology, and Chronic Inflammatory Disease Research Center, Ajou University School of Medicine, Suwon, Korea, 16499

* Correspondence: Ilo Jou (jouilo@aumc.ac.kr)

The supplementary material comprises figures S1-S7 and supplementary tables 1-2.

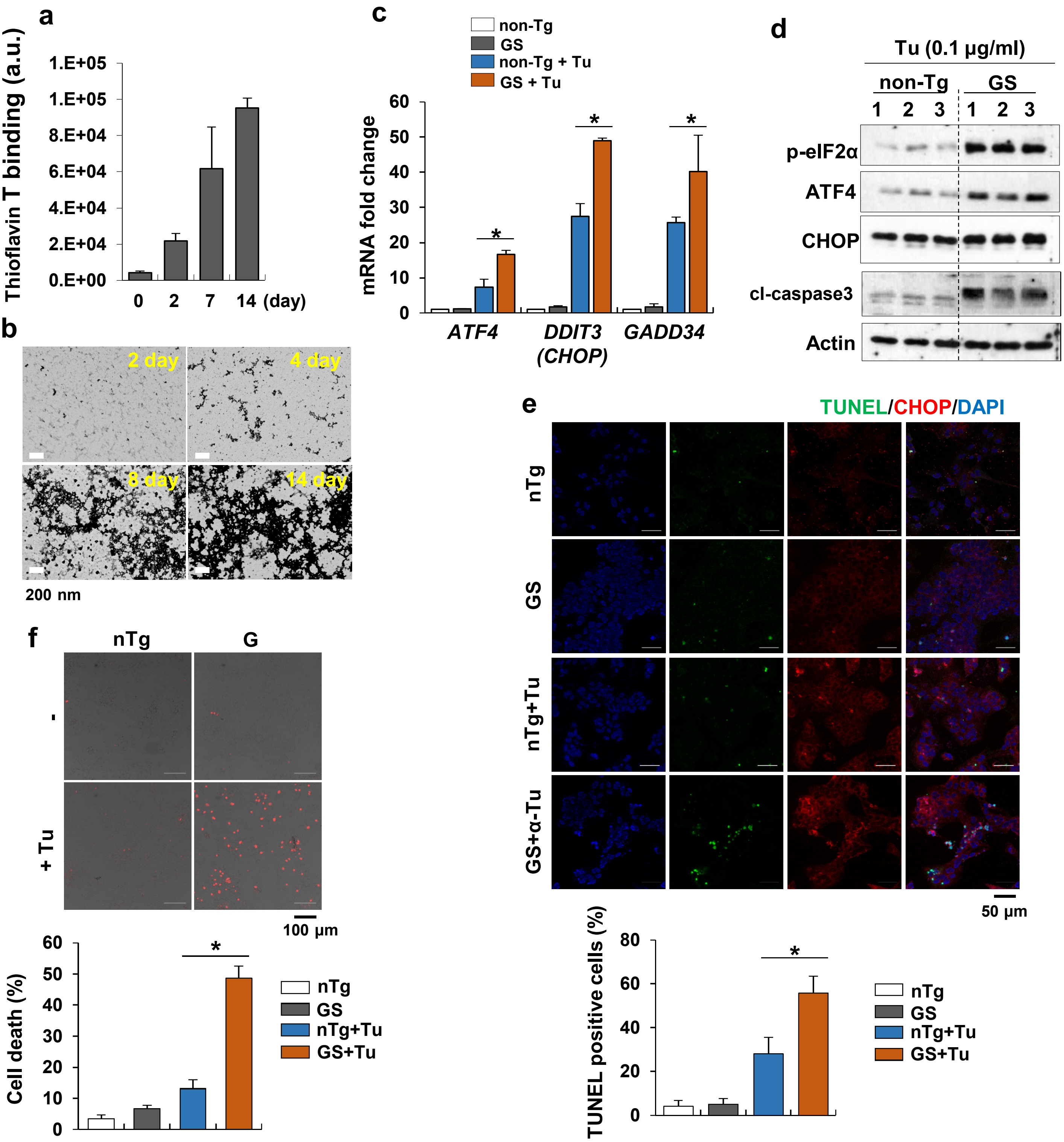

Fig. S1 The LRRK2-GS mutant induces ER stress and apoptosis in tunicamycin-treated astrocytes. Related to Figure 1

a, b Endotoxin-free recombinant human α-synuclein were incubated for 14 d. At the indicated time points, s mall aliquots were analyzed using thioflavin T binding assays (a) and observed under electron microscopy (b). **c, d** Astrocytes isolated from non-Tg (nTg) and LRRK2-GS (GS) mice were treated with tunicamycin (T u) for 24–48 h. Expression levels of the indicated mRNAs (c) and proteins (d) were analyzed by real-time q PCR and Western blotting, respectively. **e, f** Representative images and summary data showing TUNEL st aining (e) and live/dead staining (f) of non-Tg and LRRK2-GS astrocytes 72 h after treatment with Tu. All d ata are presented as means ± SD of three independent experiments (*$p < 0.05$).

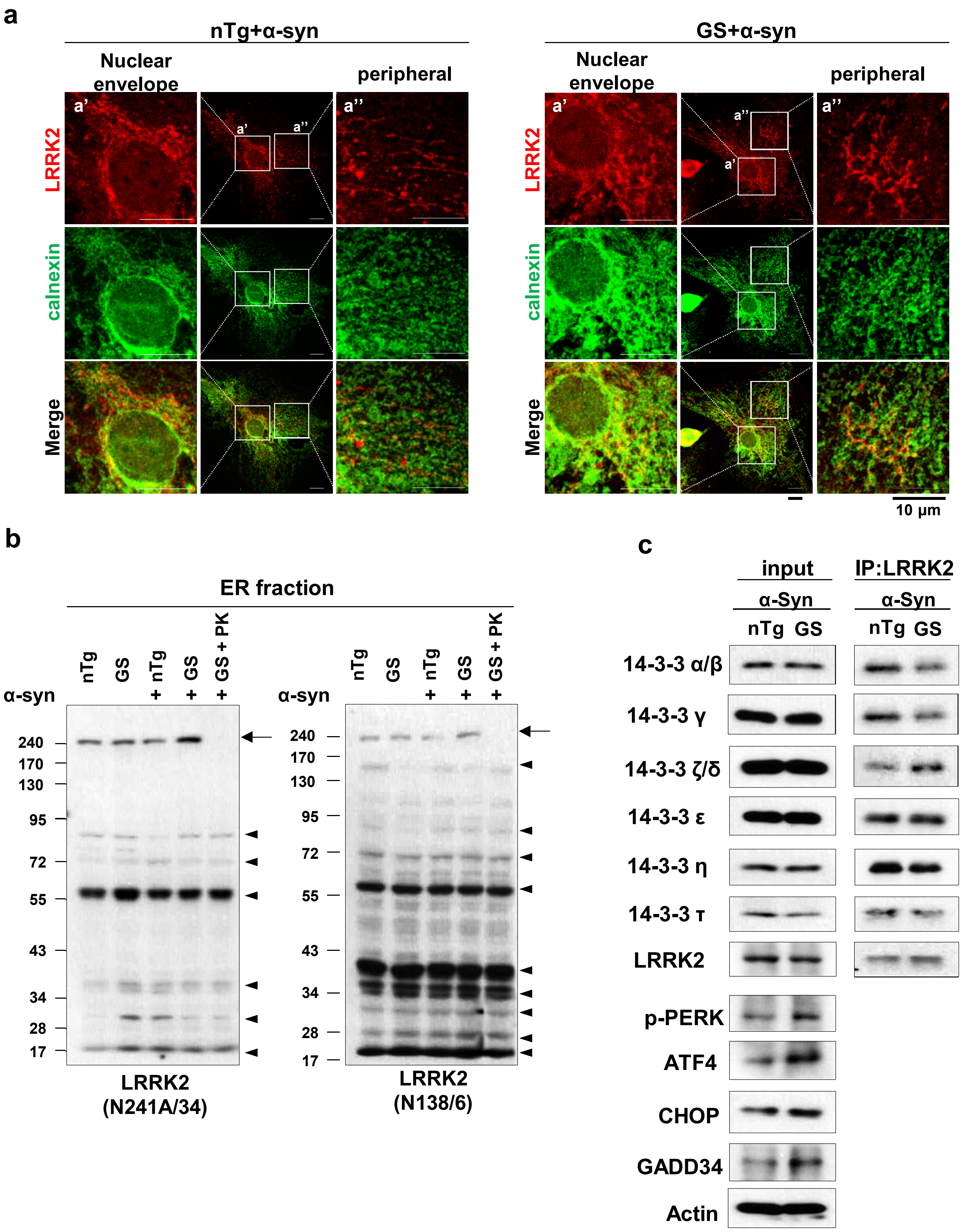

a
nTg+α-syn
GS+α-syn
Nuclear envelope
peripheral
a'
a''
LRRK2
calnexin
Merge
10 μm
b
ER fraction
α-syn
nTg
GS
+ nTg
+ GS
+ GS + PK
240
170
130
95
72
55
43
34
28
17
LRRK2 (N241A/34)
LRRK2 (N138/6)
c
input
IP:LRRK2
α-Syn
nTg GS
14-3-3 α/β
14-3-3 γ
14-3-3 ζ/δ
14-3-3 ε
14-3-3 η
14-3-3 τ
LRRK2
p-PERK
ATF4
CHOP
GADD34
Actin

Fig. S2 LRRK2-GS is localized to the ER membrane. Related to Figure 2
a Confocal images of endogenous LRRK2 and calnexin in astrocytes isolated from non-Tg and LRRK2-GS mice. Magnification of insets in A to show the localization of LRRK2 in nuclear envelope (a') and peri pheral ER regions (a''). **b** Western blot of LRRK2 in ER-enriched fractions from non-Tg and LRRK2-GS astrocytes incubated with or without proteinase K (PK). LRRK2 was detected using antibodies against th e C- or N-terminal of LRRK2 (N241A/34 and N138/6, respectively). Arrowheads indicate non-specific ba nds, and arrow indicates LRRK2. **c** Immunoprecipitation of LRRK2 in non-Tg and LRRK2-GS astrocytes, followed by Western blotting for the indicated proteins.

a

b

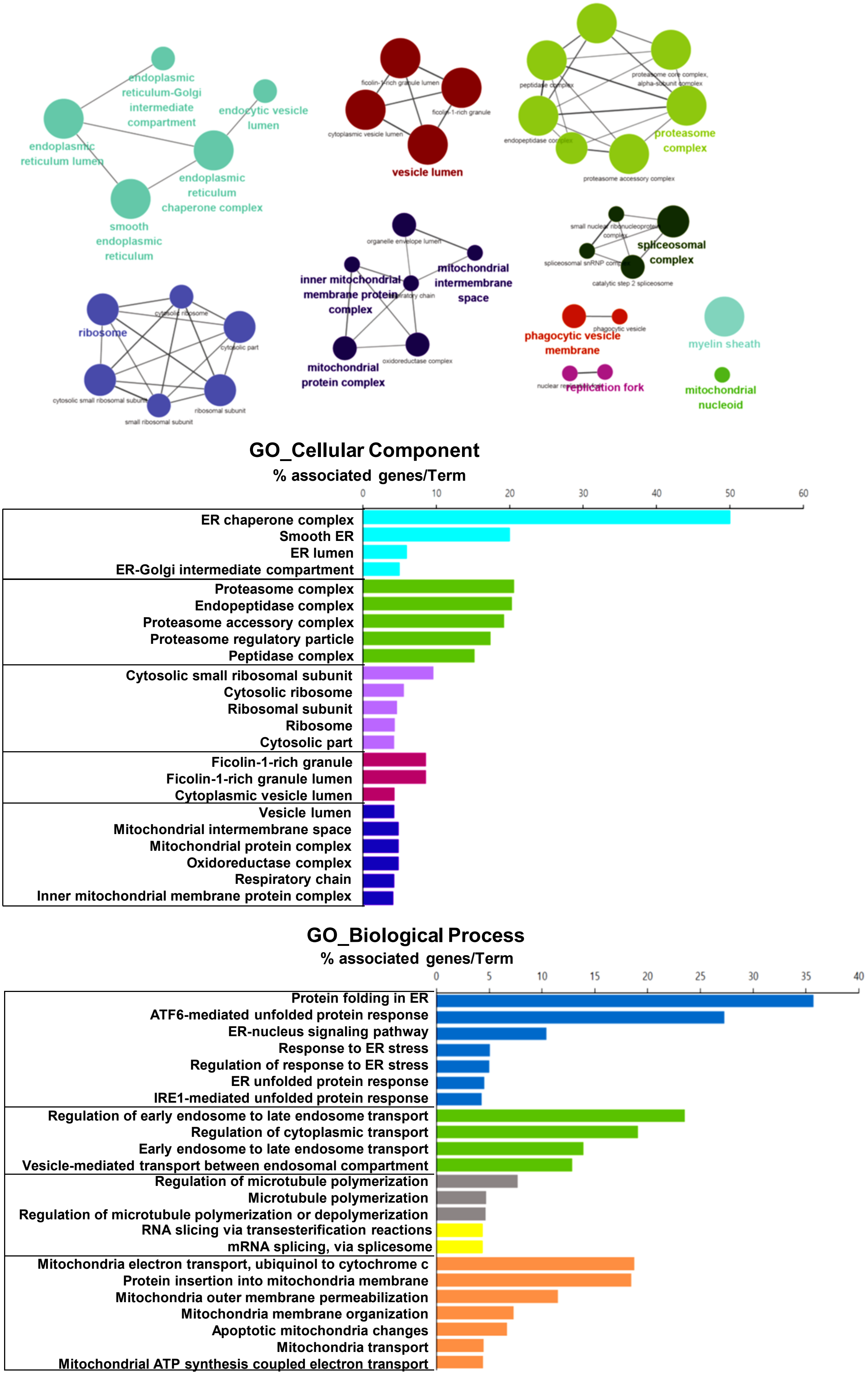

endoplasmic reticulum-Golgi intermediate compartment
endocytic vesicle lumen
endoplasmic reticulum lumen
endoplasmic reticulum chaperone complex
smooth endoplasmic reticulum
vesicle lumen
proteasome complex
ribosome
inner mitochondrial membrane protein complex
mitochondrial intermembrane space
mitochondrial protein complex
spliceosomal complex
phagocytic vesicle membrane
replication fork
myelin sheath
mitochondrial nucleoid
GO_Cellular Component
% associated genes/Term
0
10
20
30
40
50
60
ER chaperone complex
Smooth ER
ER lumen
ER-Golgi intermediate compartment
Proteasome complex
Endopeptidase complex
Proteasome accessory complex
Proteasome regulatory particle
Peptidase complex
Cytosolic small ribosomal subunit
Cytosolic ribosome
Ribosomal subunit
Ribosome
Cytosolic part
Ficolin-1-rich granule
Ficolin-1-rich granule lumen
Cytoplasmic vesicle lumen
Vesicle lumen
Mitochondrial intermembrane space
Mitochondrial protein complex
Oxidoreductase complex
Respiratory chain
Inner mitochondrial membrane protein complex
GO_Biological Process
% associated genes/Term
0
5
10
15
20
25
30
35
40
Protein folding in ER
ATF6-mediated unfolded protein response
ER-nucleus signaling pathway
Response to ER stress
Regulation of response to ER stress
ER unfolded protein response
IRE1-mediated unfolded protein response
Regulation of early endosome to late endosome transport
Regulation of cytoplasmic transport
Early endosome to late endosome transport
Vesicle-mediated transport between endosomal compartment
Regulation of microtubule polymerization
Microtubule polymerization
Regulation of microtubule polymerization or depolymerization
RNA slicing via transesterification reactions
mRNA splicing, via splicesome
Mitochondria electron transport, ubiquinol to cytochrome c
Protein insertion into mitochondria membrane
Mitochondria outer membrane permeabilization
Mitochondria membrane organization
Apoptotic mitochondria changes
Mitochondria transport
Mitochondrial ATP synthesis coupled electron transport

Fig. S3 Analysis of GS-LRRK2–interacting proteins. Related to Figure 3
a Cytoscape network analysis of LRRK2-GS–interacting proteins showing the five major Cellular Compon ent groups identified. Each group consists of nodes (Cellular Component) connected to indicate relationsh ips between nodes; each group is headed by its statistically most represented Cellular Component. **b** Iden tified LRRK2-GS–interacting proteins, grouped according to Biological Process categories.

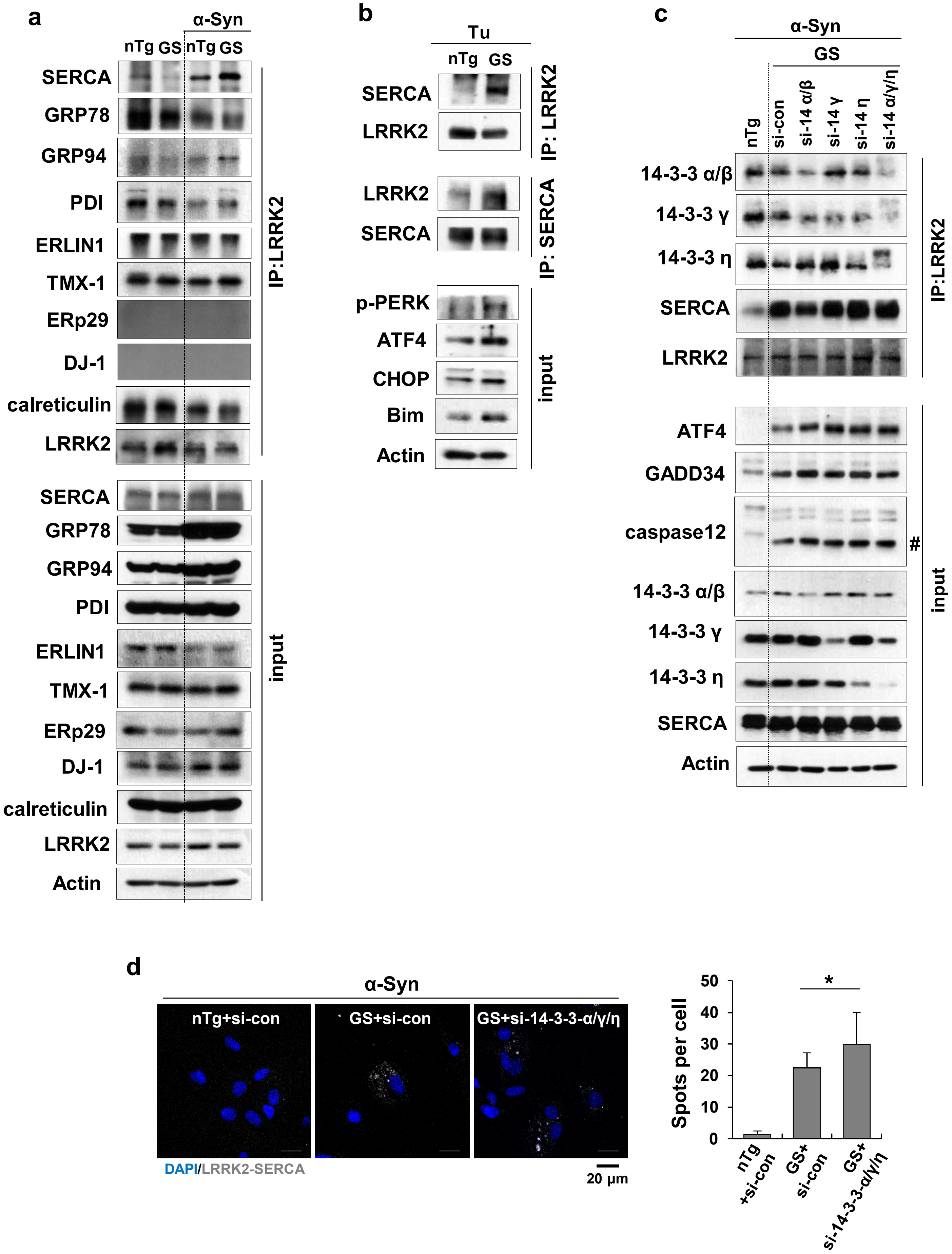
a
α-Syn
nTg GS nTg GS
SERCA
GRP78
GRP94
PDI
ERLIN1
TMX-1
ERp29
DJ-1
calreticulin
LRRK2
IP:LRRK2
SERCA
GRP78
GRP94
PDI
ERLIN1
TMX-1
ERp29
DJ-1
calreticulin
LRRK2
Actin
input
b
Tu
nTg GS
SERCA
LRRK2
IP: LRRK2
LRRK2
SERCA
IP: SERCA
p-PERK
ATF4
CHOP
Bim
Actin
input
c
α-Syn
GS
nTg
si-con
si-14 α/β
si-14 γ
si-14 η
si-14 α/γ/η
14-3-3 α/β
14-3-3 γ
14-3-3 η
SERCA
LRRK2
IP:LRRK2
ATF4
GADD34
caspase12
#
14-3-3 α/β
14-3-3 γ
14-3-3 η
SERCA
Actin
input
d
α-Syn
nTg+si-con
GS+si-con
GS+si-14-3-3-α/γ/η
DAPI/LRRK2-SERCA
20 μm
Spots per cell
50
40
30
20
10
0
*
nTg +si-con
GS+ si-con
GS+ si-14-3-3-α/γ/η

Fig. S4 Identification of GS-LRRK2–interacting proteins. Related to Figure 3

a, b Non-Tg and LRRK2-GS astrocytes were treated with α-synuclein (α-Syn) (a) or tunicamycin (Tu) (b) for 24 h and then immunoprecipitated with an antibody against LRRK2 or SERCA, followed by immunobl ot analysis with the indicated antibodies. **c, d** Non-Tg and LRRK2-GS astrocytes were transfected with si RNAs against each 14-3-3 subtype. After 24 h, cells were treated with α-Syn for 24 h. Protein interaction s were assessed by immunoprecipitation with an antibody against LRRK2 followed by Western blotting with the indicated antibodies (c); LRRK2–SERCA interactions were detected using the PLA method (d). Crosshatching denotes the cleaved caspase-12 band. PLA signals (in gray) were counted and presente d graphically as the average number of spots. Data are presented as means ± SD of three independent experiments (*$p < 0.05$).

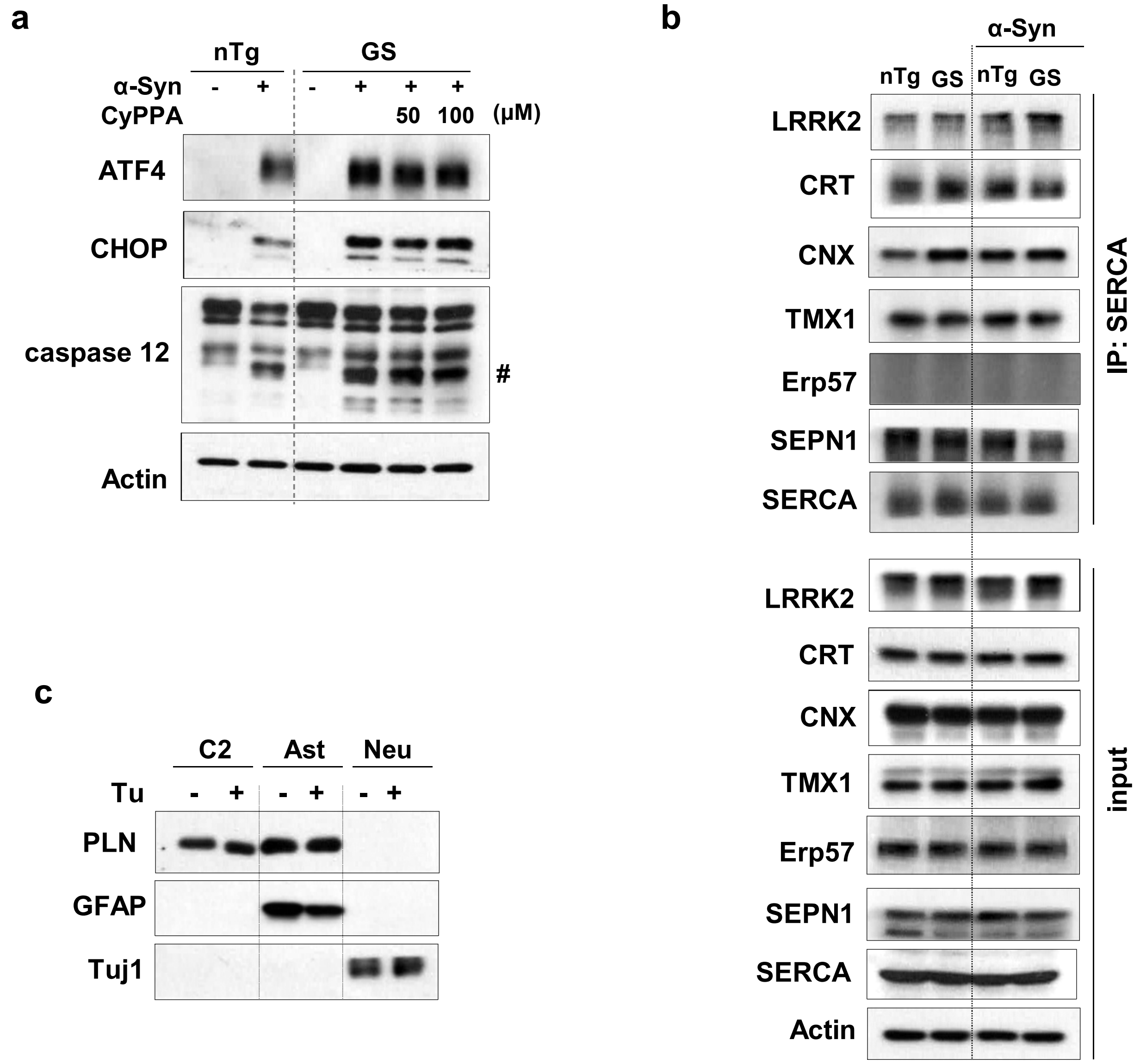

Fig. S5 LRRK2-GS promotes SERCA–PLN complex formation. Related to Figure 4

a Non-Tg and LRRK2-GS astrocytes were treated with α-synuclein (α-Syn) with or without CyPPA, a positive modulator of the SK2 channel. The indicated protein levels were analyzed by Western blotting. Crosshatching denotes the cleaved caspase-12 band. **b** Non-Tg and LRRK2-GS astrocytes were treated with α-Syn and immunoprecipitated with an antibody against SERCA, followed by immunoblot analysis with the indicated antibodies. **c** C2 myoblasts (C2), astrocytes (Ast) and primary neurons (Neu) were incubated with or without Tu for 24 h, after which levels of the indicated proteins were analyzed by Western blotting.

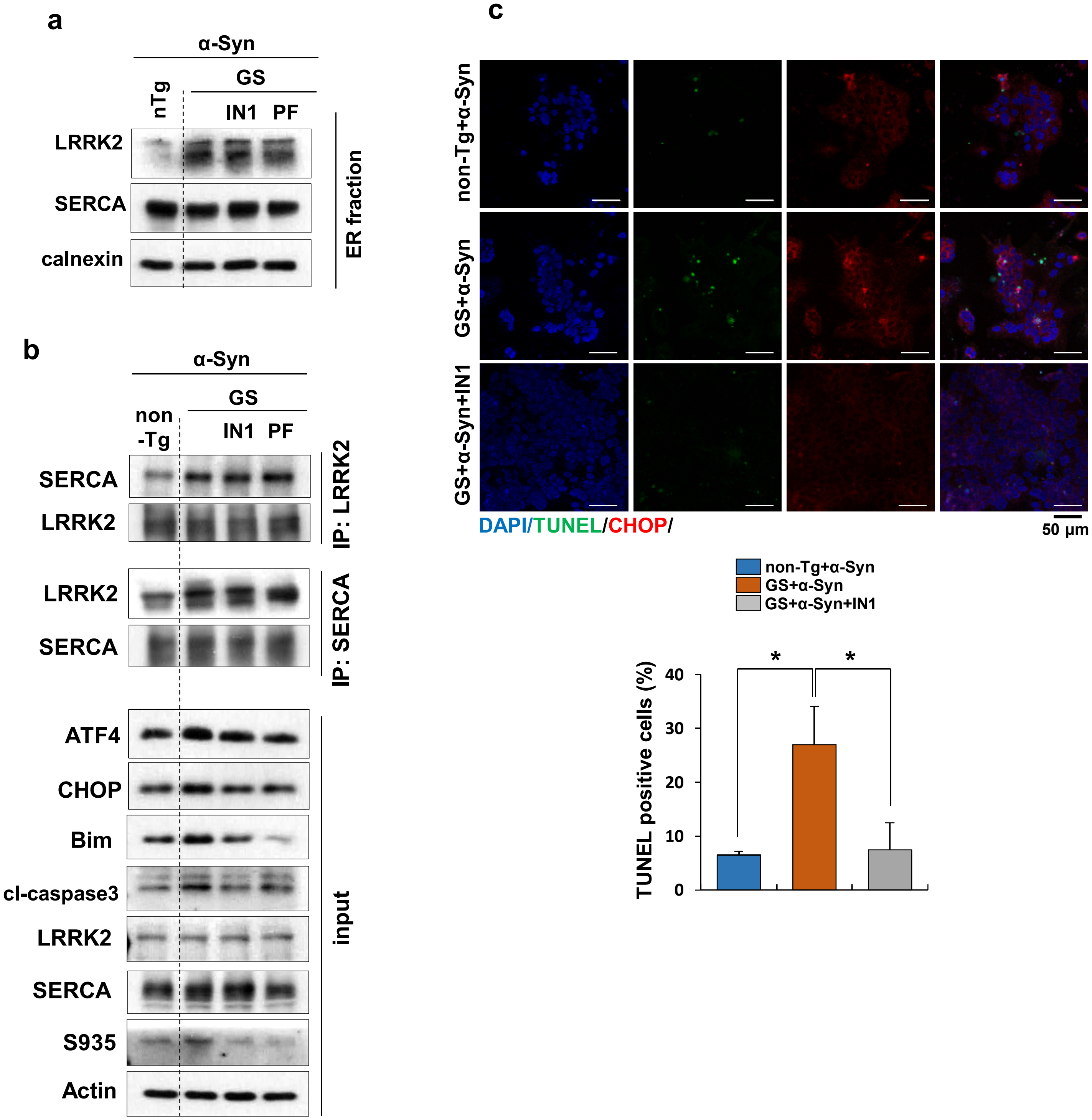

Fig. S6 LRRK2 kinase activity does not affect LRRK2 localization.
a Western blots of the indicated proteins in ER-enriched fractions from non-Tg and LRRK2-GS astrocyte s. **b** non-Tg and LRRK2-GS astrocytes were pretreated with 100 nM IN1 (Calbiochem) or 1 μM PF-06447475 (PF) (Sigma) for 10 min before α-synuclein addition. After that, cell lysates were immunopreci pitated with the indicated antibodies, followed by immunoblot analysis. **c** Representative images and su mmary data showing TUNEL staining of astrocytes isolated from non-Tg and LRRK2-GS mice 72 h after treatment with α-Syn, with or without IN1 treatment. Data are presented as means ± SD of three indepe ndent experiments (*$p < 0.05$).

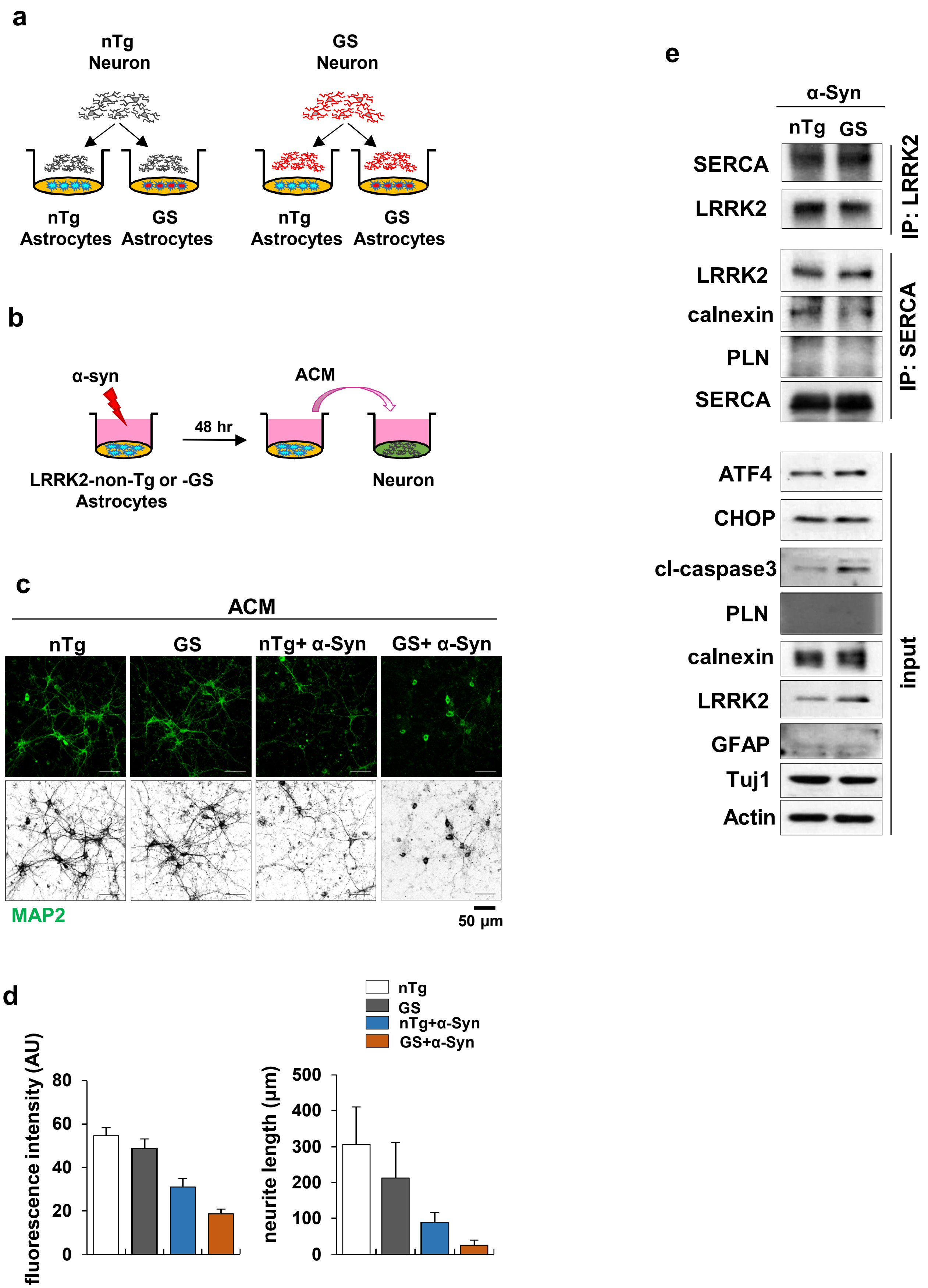

a
nTg Neuron
GS Neuron
nTg Astrocytes
GS Astrocytes
nTg Astrocytes
GS Astrocytes
b
α-syn
48 hr
ACM
LRRK2-non-Tg or -GS Astrocytes
Neuron
c
ACM
nTg
GS
nTg+ α-Syn
GS+ α-Syn
MAP2
50 μm
d
nTg
GS
nTg+α-Syn
GS+α-Syn
fluorescence intensity (AU)
80
60
40
20
0
neurite length (μm)
500
400
300
200
100
0
e
α-Syn
nTg
GS
SERCA
LRRK2
IP: LRRK2
LRRK2
calnexin
PLN
SERCA
IP: SERCA
ATF4
CHOP
cl-caspase3
PLN
calnexin
LRRK2
GFAP
Tuj1
Actin
input

Fig. S7 LRRK2-GS astrocytes affect neuronal survival. Related to Figure 6

a, b Schematic depiction of co-culture experiments (a) and neuron culture with ACM (b). **c, d** Primary neu rons were treated with ACM from α-synuclein (α-Syn)–treated non-Tg or LRRK2-GS astrocytes for 48 h. Representative immunofluorescence staining for MAP2 (c), and summary data showing fluorescence inte nsity of MAP2 per cell (n = 30 cells) and total length of neurites per cell (n = 30 cells) (d). **e** Non-Tg and L RRK2-GS neurons were treated with α-Syn for 24 h and immunoprecipitated with an antibody against LR RK2 or SERCA, followed by immunoblot analysis with the indicated antibodies. All data are presented as means ± SD of three independent experiments.

Supplementary Table 2. List of siRNA oligonucleotides

Transcripts	Target sequences
**3’UTR-LRRK2-1**	5’-GACAUCAGGCAGUCUCGAU-3’
**3’UTR-LRRK2-2**	5’-UCAGACAUCCUCGUCACUA-3’
**si-CHOP**	5’-CACGUCGAUUAUAUCAUGU-3’
*** si-14-3-3 β**	5’-CAGCUGGUAUUUGUAUCUA-3’
	5’-GUGACUAAACCCUUUACUA-3’
	5’-CAAGCCUGUCUGUAUAUCU-3’
*** si-14-3-3 γ**	5’-CGGUAGGGUUCUAAGAAGA-3’
	5’-CUGCACAUGUGACAUUGAA-3’
	5’-CGCUUGUACUGUUUGGAAA-3’
*** si-14-3-3 η**	5'-GGAGACAGUUUGCAAUGAU-3’
	5’-CUGGACUGAUGGUUGCUUU-3’
	5’-GUAACUCUUUGGCUAUUGU-3’

* : A mixture of 3 independent siRNA oligonucleotide.

Supplementary Table 3. List of primers used for qPCR

Transcripts	Forward	Reverse
**ATF4**	TCGATGCTCTGTTTCGAATG	GGCAACCTGGTCGACTTTTA
**CHOP**	GCATGAAGGAGAAGGAGCAG	CTTCCGGAGAGACAGACAGG
**GADD34**	GCTGGGTCCTTACCTTACCC	AGGGAGTGGTCACATCTTGG
**Bim**	TCCGTCTGGTATGGAGAAGG	ACATCGACACAGTGCAGAGC
**Actin**	GATCTGGCACCACACCTTCT	GGGGTGTTGAAGGTCTCAAA

Supplementary Table 4. List of primary antibodies used in this work

				Working dilution	
**Primary antibody**	**Supplier (Cat. Number)**	**RRID**	**Host**	**IB**	**IF**
Anti-phospho-PERK	Thermo Fisher Scientific (MA5-15033)	AB_10980432	rabbit	1:1000	
Anti-phospho-eIF2α	Cell signaling Technology (3398)	AB_2096481	rabbit	1:1000	
Anti-ATF4	Cell signaling Technology (11815)	AB_2616025	rabbit	1:1000	
Anti-CHOP	Thermo Fisher Scientific (MA1-250)	AB_2292611	mouse	1:1000	1:100
Anti-BIM	Abcam (ab32158)	AB_725697	rabbit	1:1000	
Anti-caspase 12	Cell signaling Technology (2202)	AB_2069200	rabbit	1:1000	
Anti-cleaved caspase 3	Cell signaling Technology (9664)	AB_2070042	rabbit	1:1000	
Anti-LRRK2	Abcam (ab133474)	AB_2713963	rabbit	1:1000	
Anti-LRRK2, C-terminus (N241A/34)	NeuroMab (73253)	AB_10671178	mouse	1:1000	1:100
Anti-LRRK2, N-terminus (N138/6)	NeuroMab (75-188)	AB_2234791	mouse	1:1000	
Anti-S935-LRRK2	Abcam (ab133450)	AB_2732035	rabbit	1:1000	
Anti-SERCA	Cell signaling Technology (4388)	AB_2227684	rabbit	1:1000	
Anti-GADD34	Abcam (ab9869)	AB_296678	goat	1:1000	
Anti-calnexin	Abcam (ab22595)	AB_2069006	rabbit	1:1000	1:100
Anti-Tom40	Santa Cruz Biotechnology (sc-11414)	AB_793274	rabbit	1:1000	
Anti-pan-14-3-3	Santa Cruz Biotechnology (sc-133233)	AB_2016726	mouse	1:1000	
Anti-14-3-3α/β	Cell signaling Technology (9636)	AB_560823	rabbit	1:1000	
Anti-14-3-3γ	Cell signaling Technology (5522)	AB_10827887	rabbit	1:1000	
Anti-14-3-3ζ/δ	Cell signaling Technology (7413)	AB_10950820	rabbit	1:1000	
Anti-14-3-3ε	Cell signaling Technology (9635)	AB_2217758	rabbit	1:1000	
Anti-14-3-3η	Cell signaling Technology (5521)	AB_10829034	rabbit	1:1000	
Anti-14-3-3τ	Cell signaling Technology (9638)	AB_2218251	rabbit	1:1000	
Anti-Grp78	Abcam (ab21685)	AB_2119834	rabbit	1:1000	
Anti-Grp94	Cell signaling Technology (2104)	AB_823506	rabbit	1:1000	
Anti-PDI	Cell signaling Technology (3501)	AB_2156433	rabbit	1:1000	
Anti-ERLIN1	Cell signaling Technology (2958)	AB_2293489	rabbit	1:1000	
Anti-TMX1	Thermo Fisher Scientific (PA5-17954)	AB_10980336	goat	1:1000	
Anti-ERp29	Abcam (ab11420)	AB_298025	rabbit	1:1000	
Anti-DJ1	Abcam (ab18257)	AB_444361	rabbit	1:1000	
Anti-Calreticulin	Cell signaling Technology (12238)	AB_2688013	rabbit	1:1000	
Anti-PLN	Abcam (ab2865)	AB_2167905	mouse	1:1000	
Anti-ERp57	Cell signaling Technology (2881)	AB_2160840	rabbit	1:1000	
Anti-SEPN1	Thermo Fisher Scientific (PA5-43082)	AB_2576858	rabbit	1:1000	
Anti-IP3R	Cell signaling Technology (3763)	AB_2129958	rabbit	1:1000	
Anti-Mfn2	Cell signaling Technology (11925)	AB_2750893	rabbit	1:1000	
Anti-cytochrome C	Santa Cruz Biotechnology (sc-13156)	AB_627385	mouse	1:1000	
Anti-β-actin	Santa Cruz Biotechnology (sc-47778)	AB_2714189	mouse	1:1000	
Anti-tubulin	Sigma-Aldrich (T6199)	AB_477583	mouse	1:1000	
Anti-Flag	Sigma-Aldrich (F1804)	AB_262044	mouse	1:1000	
Anti-Myc	Cell signaling Technology (2278)	AB_490778	rabbit	1:1000	
Anti-GFAP	Cell signaling Technology (3670)	AB_561049	mouse	1:1000	1:100
Anti-Tuj1	Abcam (ab78078)	AB_2256751	mouse	1:1000	1:100
Anti-MAP2	Abcam (ab32454)	AB_776174	rabbit		1:100
